# T Cell Transcriptomes Describe Patient Subtypes in Systemic Lupus Erythematosus

**DOI:** 10.1371/journal.pone.0141171

**Published:** 2015-11-06

**Authors:** Sean J. Bradley, Abel Suarez-Fueyo, David R. Moss, Vasileios C. Kyttaris, George C. Tsokos

**Affiliations:** 1 Division of Rheumatology, Department of Medicine, Beth Israel Deaconess Medical Center, Harvard Medical School, Boston, Massachusetts, United States of America; 2 Department of Anesthesiology, Tufts Medical Center, Boston, Massachusetts, United States of America; Nippon Medical School Graduate School of Medicine, JAPAN

## Abstract

**Background:**

T cells regulate the adaptive immune response and have altered function in autoimmunity. Systemic Lupus Erythematosus (SLE) has great diversity of presentation and treatment response. Peripheral blood component gene expression affords an efficient platform to investigate SLE immune dysfunction and help guide diagnostic biomarker development for patient stratification.

**Methods:**

Gene expression in peripheral blood T cell samples for 14 SLE patients and 4 controls was analyzed by high depth sequencing. Unbiased clustering of genes and samples revealed novel patterns related to disease etiology. Functional annotation of these genes highlights pathways and protein domains involved in SLE manifestation.

**Results:**

We found transcripts for hundreds of genes consistently altered in SLE T cell samples, for which DAVID analysis highlights induction of pathways related to mitochondria, nucleotide metabolism and DNA replication. Fewer genes had reduced mRNA expression, and these were linked to signaling, splicing and transcriptional activity. Gene signatures associated with the presence of dsDNA antibodies, low complement levels and nephritis were detected. T cell gene expression also indicates the presence of several patient subtypes, such as having only a minimal expression phenotype, male type, or severe with or without induction of genes related to membrane protein production.

**Conclusions:**

Unbiased transcriptome analysis of a peripheral blood component provides insight on autoimmune pathophysiology and patient variability. We present an open source workflow and richly annotated dataset to support investigation of T cell biology, develop biomarkers for patient stratification and perhaps help indicate a source of SLE immune dysfunction.

## Background

Systemic Lupus Erythematosus (SLE) is a debilitating autoimmune disease affecting primarily women. It involves dysregulation of T and B cells resulting in excessive production of antibodies against self proteins and DNA, immune complex formation and T cell infiltration into tissues. These processes cause a variety of symptoms including arthritis, cytopenia and kidney failure. The etiologic origins of sporadic SLE are unknown, but altered regulation of T cells is well documented [1–3]. Genetic determinates of SLE severity have been elusive in part because of the heterogeneity that marks the disease [4, 5], with the majority of cases caused by genetic predisposition coupled with environmental causes. SLE T cells present a poised activation phenotype associated with lower TCR activation threshold, lipid raft aggregation, increased calcium flux upon activation, and overproduction of inflammatory cytokines. Altered gene expression usually accompanies these functional alterations [6].

Expression signatures in SLE have been addressed primarily in the peripheral blood compartment, where pioneering work by the Pascual group first described the interferon signature [7] [8]. These genes are inducible by the cytokine in vitro and have since been subdivided as being targets of type I or II interferon [9]. Many of these are simultaneously induced in subsets of cells including T and B cells [10] and monocytes [11] providing evidence for shared signaling abnormalities in peripheral blood mononuclear cells.

We assayed steady-state mRNA abundance by sequencing to discover molecular underpinnings of T cell dysfunction in SLE. Alterations in expression reveal patient subtypes marked by induction of genes involved in protein folding on the endoplasmic reticulum, high levels of ribosomal protein genes, or the previously identified interferon signature alone. Substantial differences in T cell expression in men and women were also found. Highlighted genes could represent biomarkers informative for disease management and may also direct investigation into other T-cell driven autoimmune conditions. This methodology is amenable to study of any disease with great variability of symptom presentation if highly relevant tissue can be obtained for transcriptome sequencing.

## Materials and Methods

### Sample Collection

At least 5ml of peripheral blood was collected to Lithium Heparin BD vacutainers from 14 SLE patients under treatment at the Lupus Center at the Rheumatology Division of Beth Israel Deaconess Medical Center. All participating patients fulfilled the American College of Rheumatology criteria for the diagnosis of SLE [12]. Blood was similarly obtained from 4 similarly aged healthy female controls. This study was approved by the Institutional Review Board of Beth Israel Deaconess Medical Center. Written informed consent was obtained from all participating subjects and all clinical investigation was conducted according to the principles expressed in the Declaration of Helsinki.

### Cell extraction and RNA isolation

Rosette Sep T cell Purification (StemCell technologies, Vancouver, Canada) was employed as instructed by incubation of blood for 30 min with tetrameric antibody mixture against CD14, CD19, CD20/MS4A1, CD36, CD56, CD66b, CD123, GYPA, and CD16/FCGR3A which binds non-T cells to erythrocytes. Density-gradient centrifugation with Lymphocyte Separation Medium (Cellgro, Manassas, VA) was used to isolate the unstimulated T cells. T cell purity is routinely >93% by this method as determined by CD3 APC/Cy7 HIT3a (Biolegend) staining detected on a Beckman Coulter Gallios Cytometer. RNA was then prepared by Qiagen AllPrep Kit (Valencia, CA) from 3 million T cells with DNAse-I treatment. Roughly 2ug of total RNA was submitted to sequencing, and OD260/280 ratios were approximately 2.1.

### Sequencing and Analysis

Unstranded cDNA library preparation and sequencing was performed by BGI (Shenzhen, China). Illumina sequencing provided roughly 75e6 paired 90bp reads for each (~ 12GB gzipped data per sample), which were assessed by FastQC and trimmed to allow ~85% mapping to the GRCh37/hg19 assembly by TOPHAT. Expression scores in Fragments Per Kilobase of transcript per Million mapped reads (FPKM) were obtained by CUFFLINKS for the 24262 best annotated genes, including many expressed psuedogenes and noncoding RNAs (S1 Table). For comparisons between different groups of samples, CUFFDIFF2 was used primarily, alongside DESeq2 and nonparametric tests in R, to calculate expression and statistical differences. Heatmaps were generated using median-normalized expression data with Gene-e and NMF clustering was performed on the Genepattern server, both provided by the Broad Institute. Singular Value Decomposition (SVD) was performed at http://biographserv.com/ and Venn diagrams were created with BioVenn [13]. Online supplemental files contain methods with specific program commands and R scripts which were implemented in R studio (S1 Text).

## Results

**Fig 1:**
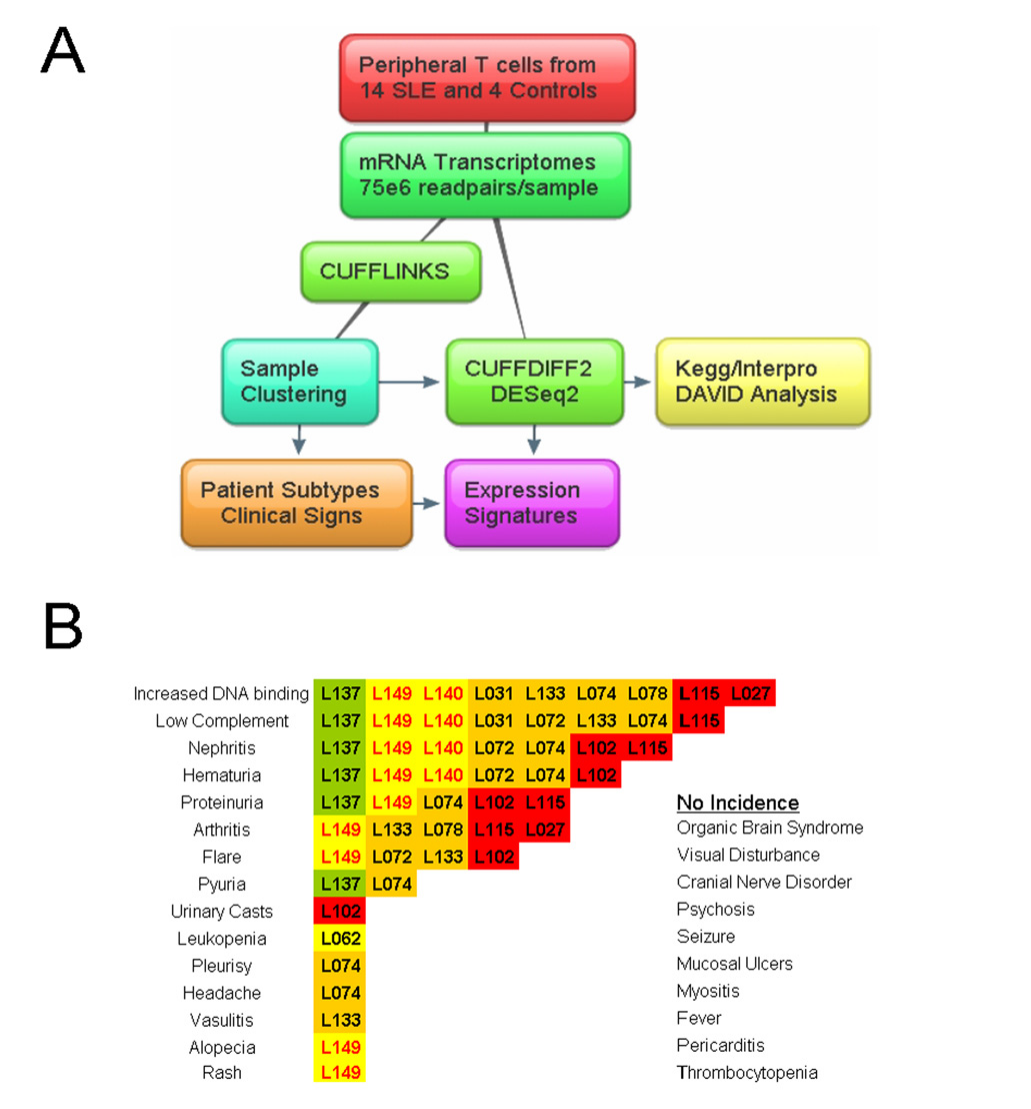
T cell transcriptome workflow and distribution of major clinical signs in the SLE patient cohort. **A)** Samples were obtained from peripheral blood by negative selection and mRNA was sequenced at high depth. CUFFLINKS generated per-sample expression values and CUFFDIFF2 and DESeq2 were used for groupwise comparisons, which were repeated following discovery of novel sample subgroups. **B)** Frequency of SLE symptom presentation at blood draw for patient samples. Highlighting colors based on patient subtypes determined by downstream analysis.

We sequenced mRNA from peripheral T cells in two men and 12 women with a variety of SLE manifestations and SLEDAI disease scores (Fig 1) [14]. As controls we prepared specimens from 4 healthy women aged 25 to 37. None of the patients were receiving therapy with biological agents (Table 1). To evaluate the cell-type purity of the samples we checked non-T cell marker expression. Of the epitopes used to collect T cells by rosette negative selection, only one had expression above background. CD16/FCGR3A, a receptor on NK and T cells [15], had medium expression and was induced in some patients relative to controls. Negligible *CD5* and *CD19* signal indicated that the preparation was free from B cells and our T cell purity is routinely >93% based on surface CD3 detection. Genes were stratified by expression (average value of all 18 samples) into classes of high, medium, low and unexpressed (≥34, ≥11, ≥1 and less than 1FPKM). Most analysis was carried out on the top quartile of expression in the genome (6047 genes) which included high and medium classes. Among the highest expressed were B2 microglobulin (*B2M*), several thymosins and the expected myriad ribosomal and mitochondrial proteins.

**Table 1:** Clinical manifestations of the enrolled SLE patients.

ID	L005	L137	L101	L062	L149	L140	L031	L072	L133	L074	L078	L102	L115	L027
Subtype	0	0	0	A	A	A	B	B	B	B	B	C	C	C
SLEDAI	0	16	0	1	20	8	4	6	16	26	6	12	12	6
Race	white	white	white	black	white	asian	white	black	white	black	white	white	asian	white
Sex	F	F	F	F	M	M	F	F	F	F	F	F	F	F
Years	55	39	40	32	33	30	43	39	51	31	61	24	28	48
Low Complement		yes			yes	yes	yes	yes	yes	yes			yes	
Increased DNA binding		yes			yes	yes	yes		yes	yes	yes		yes	yes
Arthritis					yes				yes		yes		yes	
Hematuria		yes			yes	yes		yes		yes		yes		
Proteinuria		yes			yes					yes		yes	yes	
PGA	1	2	0	0	2	0	1	2	3	2	1	2	3	1
C3 (mg/dl)	137	73	99	92	128	89	89	72	91	51	91	115	73	90
C4 (mg/dl)	13	7	24	23	7	34	17	7	2	11	11	25	17	18
dsDNA	neg	(1:40)	neg	neg	(1:320)	(1:20)	(1:40)	neg	(1:80)	(1:80)	(1:80)	neg	(1:80)	(1:320)
ESR	20	12	9	15	10	49	38	39	40	120	38	17	65	5
WBC/HPF	1	57	0	0	8	0	3	0	1	6	0	2	3	0
RBC/HPF	1	125	0	0	12	8	0	175	1	28	0	23	1	0
Urine Protein	30	300	0	30	30	30	0	x	0	1566	100	100	245	0
Spot/creatinine	0.8	1.9	0.1	0.2	x	0.2	0.1	0.8	0.3	4.6	1.1	1.9	3.6	x
WBC	6	8	6	3	4	8	6	9	8	8	6	9	13	4
Lymphocyte	1298	1957	1112	1079	954	832	1224	3355	600	1771	1462	335	1454	752
Hemoglobin	12	11	13	11	14	13	13	11	13	12	13	10	10	12
Platelets	146	320	259	191	238	283	336	273	279	312	241	312	352	220
Creatinine	0.6	0.7	0.5	0.5	0.8	1	0.9	0.9	0.6	0.5	0.9	1.2	2.3	0.6
Prednisone (mg)	0	60	0	0	0	10	10	30	20	40	0	40	10	4
Hydroxychloroquine	400	0	400	400	400	200	400	0	0	0	400	400	0	400
Azathioprine	100	0	0	0	0	0	100	0	0	0	0	0	0	0
Mycophenolate	0	0	0	1000	0	1500	0	3000	0	3000	750	0	2500	1500

Increased dsDNA binding (dsDNA), low complement (C3 or C4) or nephritis (hematuria or proteinuria) were the major symptoms most prevalent. SLEDAI, Systemic Lupus Erythematosus Disease Activity Index; PGA, Physician's global assessment; ESR, erythrocyte sedimentation rate; WBC, white blood cell; RBC, red blood cell; HPF, high-power field. Nephritis was confirmed by recent biopsy and patient L078 has minimal change disease deemed unrelated to lupus.

Although total T cells are readily obtained and efficiently purified, variable numbers of constituent cell types impacts transcript abundance. Our pan-T cell view provides breadth and flexibility for study but does not address alterations in cell type frequency, such as lower total lymphocyte counts or lower proportion of CD4 (fraction and absolute amounts) often found in SLE patients. Genes with significant differential expression usually had greater than 2-fold changes in transcript signal, and therefore likely reflect expression changes more so than differences in cell type frequency associated with SLE.

### SLE T cells display more genes with increased rather than decreased expression

**Fig 2:**
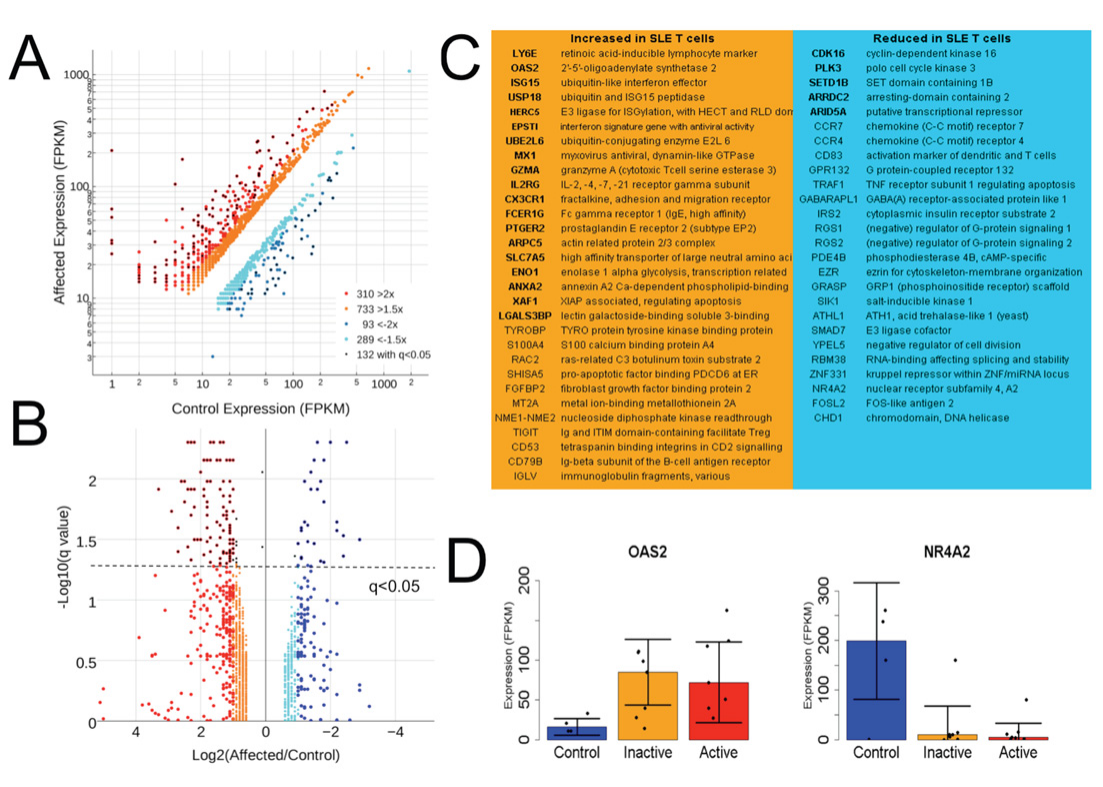
SLE T cells display more genes with increased rather than decreased expression. **A)** Distribution of expression stratified at the 1.5-, 2-fold and q<0.05 CUFFDIFF2 significance levels. **B)** Relationship between q values and expression fold change in SLE relative to control. **C)** Select genes significantly increased or decreased as determined by sequencing and CUFFDIFF2, with those confirmed by DESeq2 in bold. **D)** Example constituent data for OAS2 and NR4A2 in control, inactive and active (SLEDAI >6) samples, where error bars represent the median absolute deviation about the median.

First we sought an overall picture of mRNA abundance changes related to SLE in T cell samples. Differential expression metrics were found by CUFFDIFF2, which calculates groupwise expression in Fragments Per Kilobase of transcript per Million mapped reads (FPKM) to allow comparison across genes of varied size and applies a beta negative binomial distribution to generate a False Discovery Rate (FDR) where q<0.05 is routinely considered significant [16]. A scatterplot shows the distribution of fold change by expression level for genes with a 1.5- or 2-fold difference and those detected as significantly altered (Fig 2A). Count-based methods of differential expression are reported to be advantageous in some scenarios, so we also used DESeq2 which was usually confirmatory (p<0.01). Alterations were stratified at multiple levels to provide flexibility for downstream analysis, some of which performed better with more input genes having more subtle differential expression. For high- and medium-expression genes (6047 with greatest average expression for all samples) at least twice as many displayed increased rather than reduced mRNA expression relative to controls at all thresholds. Alterations at each threshold were similarly distributed in terms of mRNA abundance. One third of the genes showing more than 2-fold expression changes were statistically significant by CUFFDIFF2 (q<0.05) but only one third of these passed a comparable threshold in DESeq2 (p<0.01) (Fig 2B).

Previously reported interferon signature genes including *OAS2*, *ISG15*, *UBE2L6*, *IFI35*, *IFI44*, and *STAT1* were detected as significantly induced by both analyses [17]. Among highly-expressed and significantly upregulated genes were *IL2RG* (encoding CD132, the common subunit of receptors for Il-2, -4, -7–15 and -21), *CD53*, *ENO1* and many immunoglobulin fragment transcripts. Select genes with diverse functions are listed, with those in bold found by both CUFFDIFF2 and DESeq2 (Fig 2C). Genes with significantly reduced mRNA abundance included *RGS1* and *RGS2*, which drive G-protein alpha subunits to their inactive state, *EZR*, which regulates cytoskeleton-membrane interactions, and several nuclear expression regulators. *OAS2* and *NR4A2* serve as examples of robust alterations, which usually persisted in patients both with low and high (SLEDAI >6) disease activity (Fig 2D), but were not altered in all patients. Although *CCR4* and *CCR7* mRNAs were significantly reduced, their surface expression is reported to be increased on SLE T cells [18, 19], suggesting altered post-translational or membrane trafficking regulation for these receptors.

### Genes Related to Disease Symptoms

Gene expression markers of SLE symptoms could aid in diagnosis and may point to causative biology. We sought expression changes linked to the presence of increased anti-dsDNA antibodies, low complement levels, or nephritis. Comparison to controls largely recapitulated the overall SLE analysis, showing similar gene expression related to all major symptoms. This was not surprising given that many patients exhibited more than one symptom. We therefore made comparisons among patient samples with and without each SLE manifestation. Although CUFFDIFF and DESeq analyses suggested genes with substantial fold changes and statistically significant differences in expression, the underlying data revealed great vulnerability to outlier expression. We employed the Mann-Whitney nonparametric rank-sum test, based on groupwise median rather than mean expression values, which yielded genes with statistically different expression in samples with and without each clinical sign.

**Fig 3:**
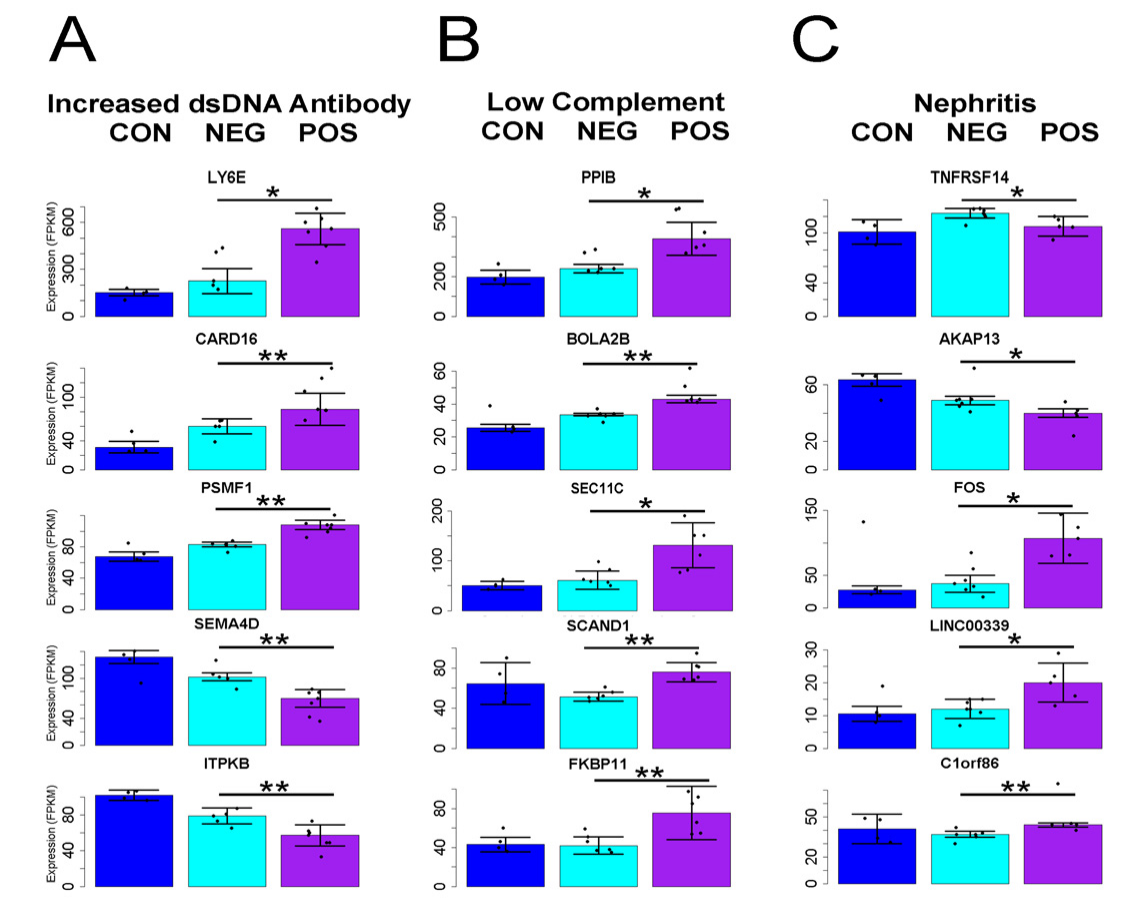
T cell expression of specific genes linked to major clinical manifestations of SLE. Select genes with differential expression in women largely unique to each symptom were detected by Mann-Whitney rank sum tests related to **A)** increased presence of dsDNA antibodies **B)** Low C3 or C4 complement levels or **C)** biopsy-confirmed lupus nephritis. Error bars represent median absolute deviation from the median value for each group and *p<0.05, **p<0.01 for pairwise tests conducted on samples from patients with or without each symptom (healthy control data plotted only for comparison).

Samples obtained from patients with increased dsDNA antibodies (titer greater than 1:40 at blood draw) had 579 and 44 genes detected as significant at the p>0.05 and 0.01 levels, respectively (Fig 3A). Most compelling was confirmation of an association with increased expression for *LY6E* [20]. More mRNA for caspase inhibitor *CARD16* and proteasome regulator *PSMF1* was also detected. Reductions were evident for mRNA for *SEMA4D*, which has altered expression in arthritis [21], and *ITPKB*, encoding an inositol phosphate kinase involved in stem cell division [22, 23]. DESeq2 confirmed the association of all but *PSMF1* with this clinical feature (p<0.01), while CUFFDIFF2 confirmed only *LY6E*. None of the genes associated with other symptoms were confirmed by either analysis.

A similar number of gene expression differences uniquely marked samples obtained from patients having low complement (C3 lower than 90 mg/dl or C4 less than 12 mg/dl). Most of the 388 and 46 genes detected at the p<0.05 and <0.01 levels had increased expression (Fig 3B). Several of these have activity at the endoplasmic reticulum. *SEC11C* encodes a subunit of microsomal signal peptidase complex while peptidyl-prolyl cis/trans isomerases cyclophilin B (*PPIB*) and *FKBP11* both support protein folding. Increases in *BOLA2*, which binds glutaredoxin 3 to regulate iron levels [24] and the poorly characterized *SCAND1* are also good candidate genes for which increased activation marks this clinical phenotype. The function of these genes hints at a disruption of normal activity rather than increased T cell activity.

Patients with nephritis (confirmed by recent biopsy and usually coincident with proteinuria or hematuria) showed fewer genes with altered expression (58 or 2 for p<0.05 or 0.01) and there were no obvious links between them (Fig 3C). The coefficient of variability for altered genes (p<0.05) was greater for these samples (1.2 versus 0.9 and 1.1 for DNA antibodies and low complement, respectively). Patient L078 biopsy and electron microscopy indicated minimal change disease unrelated to lupus, and exclusion from the non-nephritis group had no effect on the detected signature genes because non-parametric tests are only mildly affected by single sample values. One striking marker was *TNFRSF14/HVEM*, encoding a coreceptor for herpes virus which transduces immunosuppressive signals from BTLA [25]. Although this mRNA was marginally increased in SLE patients relative to controls, patients with nephritis had significantly lower amounts relative to those without. A similar pattern was found for many genes increased in SLE, including *OAS2* and *MX1*, which may indicate progression of disease beyond functional immune signaling within T cells and on to response to renal breakdown. Other increased mRNAs linked to the presence of nephritis were *C1orf86/FAAP20*, coding for a DNA repair factor [26], and *LINC00339*, an uncharacterized noncoding RNA. *FOS* mRNA was strikingly increased. This member of the AP1 transcription complex has numerous immune roles, and mRNA for its paralog *FOSL2* was among those significantly decreased overall in patient samples.

Although we detected mRNAs marking the presence of major clinical signs of SLE, the fold changes were less than expected and the genes involved did not suggest a clear picture of the relationship between T cell expression and cause of the symptoms. This could be due to the small size of our cohort and the fact that multiple overlapping symptoms were present in several patients.

### Novel Patient Subtypes are Detected by T cell Expression Clustering

Next, we looked for expression patterns among all patients that might uncover subgroups. We applied unbiased clustering (Pearson) to organize samples by expression similarity in a heatmap, first using genes with at least 1.5-fold expression changes in SLE versus controls. Initial clustering was skewed by outlying expression in single samples. Though potentially interesting they disrupt visualization of groups with coherent behavior. To purify the data we filtered for genes with coefficients of variation (Standard deviation divided by average) between 2 and 0.3, which yielded roughly 1000 genes.

**Fig 4:**
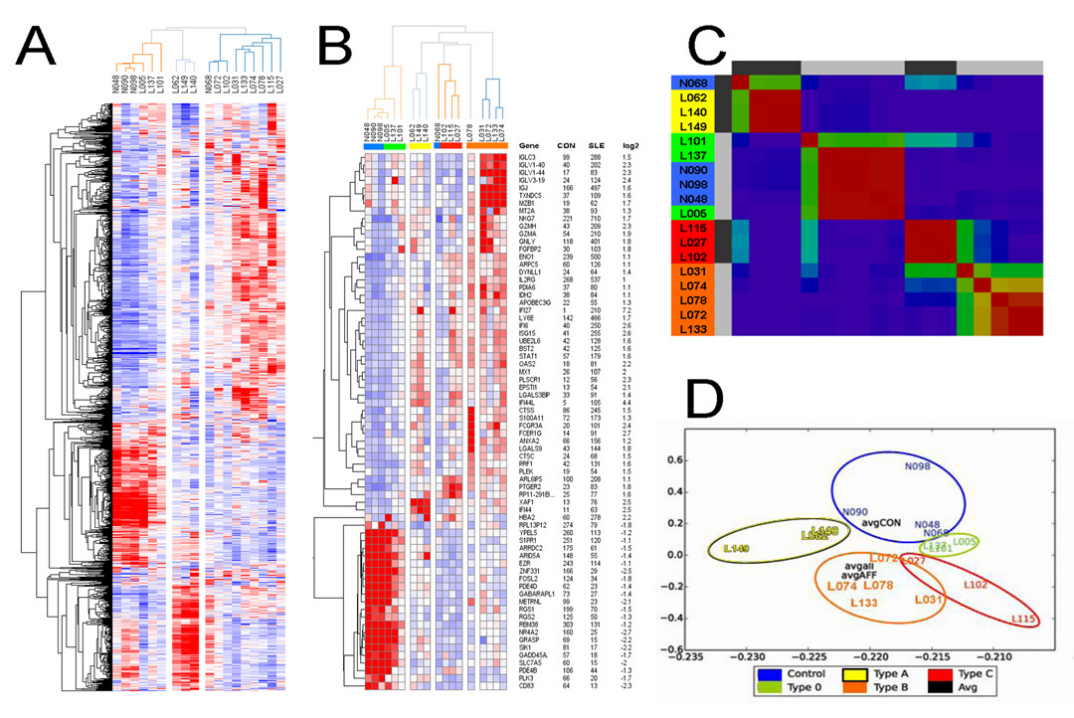
Unbiased clustering identifies patient subtypes by T cell gene expression. **A)** Genes with altered expression in lupus T cells relative to controls, which also showed moderate variation across all samples, were median normalized and Pearson clustered with average linkage, where red and blue denote high and low expression. **B)** Removal of genes with expression outside of the top quartile permits identification of subtype signature genes. **C)** Unbiased NMF clustering using the same input genes yields similar patient subgroups. **D)** SVD clustering of samples provides an approximate metric of sample similarity, where average values for control, affected and all samples are centrally located.

Immediately obvious was that three SLE samples cluster with the controls, indicating similar T cell gene expression (Fig 4A). These samples exhibit only a minor T cell expression phenotype we term Type 0. Two of these patients had low disease activity (SLEDAI 0), but L137 had a higher disease score so its control-like expression pattern is surprising, and may be explained by high dose prednisone treatment. Two other sample groups were delineated by high expression for two different sets of genes (red in the bottom middle for three sample columns, or more diffusely in the upper right for nine samples). The middle group contains samples from two men with active disease and a woman (L062) with SLEDAI 1, which we denote Type A. This unbiased organization of samples is striking because active and inactive samples cluster together, uncoupling disease score from T cell expression in some cases.

To view modules with common patterns we repeated clustering following removal of entries with lower than top quartile expression. This made additional sample types apparent among those from SLE patients (Fig 4B). At the top of this heatmap is a module of genes induced in four patients with increased immunoglobulin fragment mRNAs as well as two genes whose products act at the endoplasmic reticulum, peptide isomerase chaperone *TXNDC5* [27] and *MZB1*, which promotes IgM assembly. Another module specific to three patients was marked by induction of *ARL6IP5/JWA*, an ROS-sensitive ER protein involved in DNA repair, prostaglandin receptor *PTGER2* and an expressed pseudogene of ribosomal protein *RP11*. We denote these sample groups as Type B and C, and they were similar both in the cohort of genes and extent of expression change outside of these striking modules. They present a more severe expression phenotype than the other sample groups, but most of the alterations are detectable to a minor degree in Type 0 patient samples. In this view, outlier expression for L078 is readily identified. The induction of several genes unique to individual samples was pervasive in our cohort, and may hint at a common disease mechanism linked to transcriptional regulatory failure.

Membership in each group was somewhat dependent on the algorithm and expression thresholds employed, but the four types of patient samples persisted across clustering schemes. We used other methods to verify the tendency to form these groups. Non-negative matrix factorization (NMF) largely recapitulated the Pearson clustering (Fig 4C) and helped confirm that L078 was most similar to Type B, although it represents an edge case. Singular Value Decomposition (SVD) organization of the samples provided additional evidence for structured similarity, where Type 0 grouped with the controls, Type A is quite separate, and Types B and C are closer together (Fig 4D). In each case Type 0 samples were positioned between the controls and Types B and C, indicating an intermediary or perhaps transitional expression phenotype. We repeated CUFFDIFF analysis to find genes altered in each sample subtype with regard to the controls (S1A Fig) and found more alterations linked to Type B and C, consistent with the segregation resulting from unbiased clustering. Most of the altered expression found in Type 0 was also detected in Type B and C samples. Type B had a greater number of genes that were different from all other sample types (S1B Fig). The clinical data associated with these sample groups did not show striking patterns, other than the fact that Type B and C samples were obtained from patients with more symptoms and higher disease scores (orange and red highlighting, Table 1).

**Fig 5:**
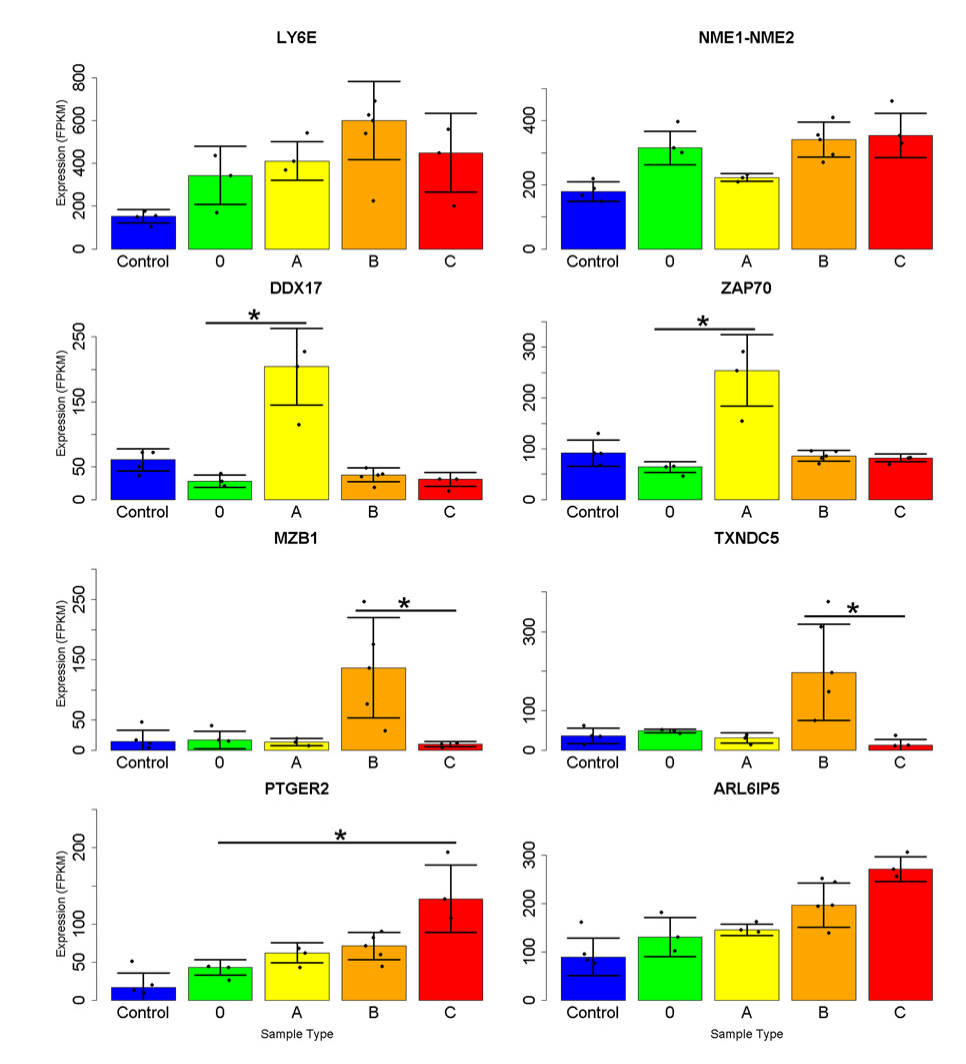
Marker gene expression suggested to identify SLE patient subtypes. Genes were selected based on their ability to differentiate first all SLE samples from controls (top) and then subtypes A or B from the others. Although not all of the genes selected had statistically different expression from all other groups, their use in concert is expected to be sufficient for stratification. Error bars represent median absolute deviation from the median value for each group, and * signifies p<0.05 by Kruskal-Wallis rank-sum followed by Bonferroni-corrected Dunn post test.

We next sought expression markers capable of partitioning samples into the patient subgroups. CUFFDIFF and DESeq comparisons yielded largely overlapping lists of genes, similar to our findings related to potential mRNA biomarkers of SLE clinical manifestation. Although many genes have significant expression alterations in SLE T cells, most are driven by differences present in less than half of the patients in our cohort due to the relatively mild expression phenotype samples in subgroup 0 and A. We applied the nonparametric Kruskal–Wallis and Dunn tests, which allows for comparison between multiple groups. Putative markers were then prioritized by specificity and high expression (Fig 5). An exception was Type 0 samples, for which *LY6E* and *NME1-NME2* expression was chosen on account of the induction present in all subtypes relative to controls. Type A samples exhibited high levels of *DDX17* and *ZAP70* mRNA, while type B samples show increased *MZB1* and *TXNDC5* are expected to best differentiate them from type C samples. Type C samples were less obviously marked but displayed higher levels of *PTGER2* and *ARL6IP5* mRNA compared to Type A.

Immunosuppression is a serious confounder of human autoimmunity studies, so we also looked for expression differences related to prednisone use. CUFFDIFF and DESeq detected relatively few significant alterations (88 and 4 respectively at q<0.05 and p<0.01), for which only *TXNDC5* and *MZB1* had been highlighted as related to patient status or subtype. We next looked among the 324 genes with at least 1.5-fold expression change in patients with or without prednisone administration for genes of interest in other comparisons. This more liberal view revealed *FOS* and *LINC00339* (induction linked to nephritis), as well as *MZB1* and *TXNDC5* (induced in Type B patient samples), as increased in patients under steroid treatment. Mann-Whitney analysis detected 139 genes at the p<0.05 threshold, of which only *FOS*, *LINC00339*, and *C1orf86* had been previously noted (all increased in nephritis). These results indicate that prednisone may underlie expression that we found related to nephritis or Type B status. There is considerable overlap for these attributes in our cohort, especially for nephritis and prednisone use. However, based on relatively high expression of Type B markers even for patient L078, who was steroid-free, we expect confounding effects to be ruled out in future studies. We suspect patient L137 to be present in the mild phenotype subgroup on account of high dose prednisone treatment.

### Functional Annotation detects Pathways and Protein Domains Linked to SLE Expression Changes

**Fig 6:**
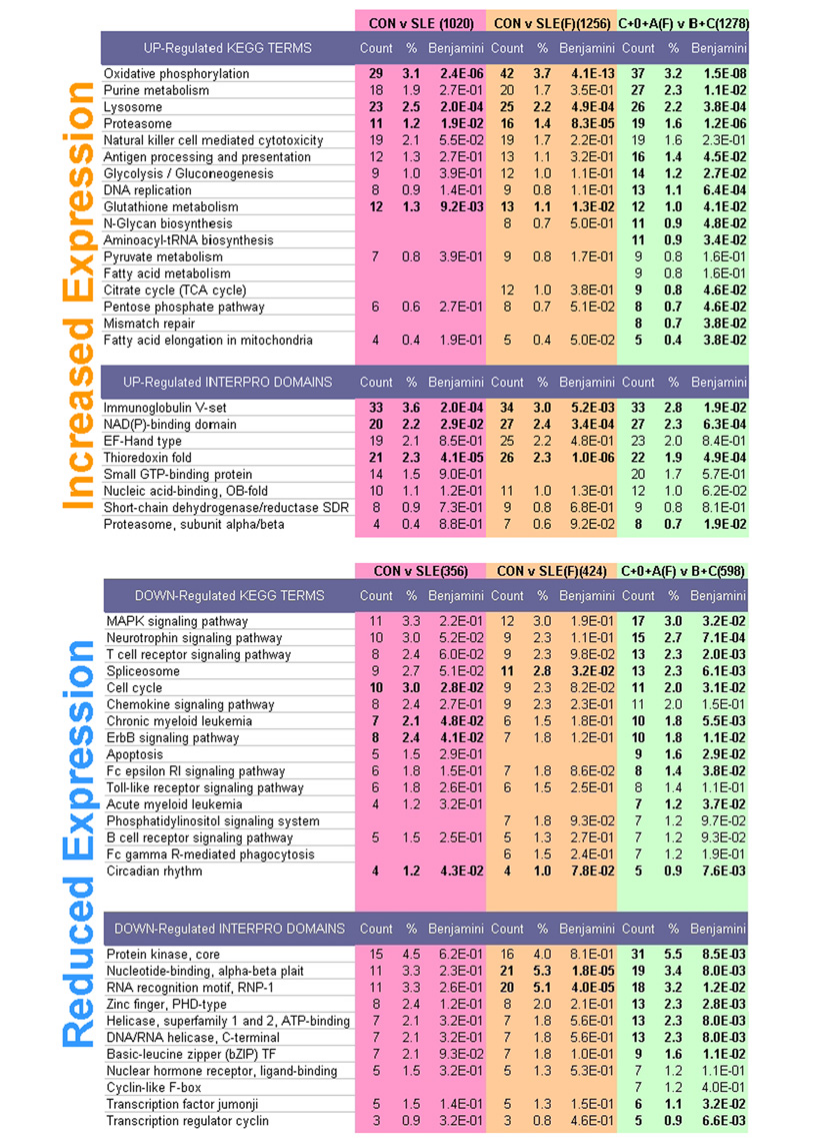
Distinct biological pathways and protein domains are identified following sample clustering. The number of genes with altered expression used for each query is in parentheses for each comparison. Significant terms with Benjamini q values <0.05 are in bold. More terms were detected for Control versus SLE samples by removal of male samples (CON v SLE(F)). Further refinement was obtained by grouping minor expression phenotype samples instead with controls (C+0+A(F) v B+C). Redundant and nonspecific terms were discarded and the remaining were ranked by the number of genes associated.

DAVID analysis provides a literature-based overview of biological functions related to differential expression [28], where KEGG pathways and INTERPRO protein domains offer concise and non-redundant terminology. We compared SLE samples to controls, and also pooled controls with mild expression phenotype samples (Type 0 and A females) for comparison with grouped Type B and C samples. Patient subtype information strengthened this analysis because the comparison of samples with weak or strong expression phenotypes detected more significant terms, and more genes associated with each, than did the initial control versus SLE analysis (Fig 6).

For genes induced in SLE, significantly related KEGG pathways are readily associated with activated and proliferating immune cells, and included Oxidative Phosphorylation (37 genes), Lysosome (26), Proteasome (19) Antigen Processing (16), Glycolysis (14), N-Glycan biosynthesis (11) and Fatty Acid Elongation (5). Significant INTERPRO terms included Immunoglobulin (33) and NAD(P) Binding (27) domains. Down-regulated genes were associated with signaling and nuclear pathways including Spliceosome (13 genes), FC receptor RI Signaling (8), Erb Signaling (9) and Apoptosis (9) and Circadian Rhythms (4). Genes with reduced expression in SLE were enriched for domains related to signaling and gene expression, including Kinase (31), Zinc-Finger (13), Basic Leucine Zipper (9) and Jumonji Transcription Factor (6) motifs. The genes related to each term are listed in the supplement (S2 Table). Analysis of patient subtype samples did not offer compelling differences from these ontology terms, presumably because fewer genes were specific to each.

### Induced Transcription Factors Are Suggested to Regulate Induced Genes

**Fig 7:**
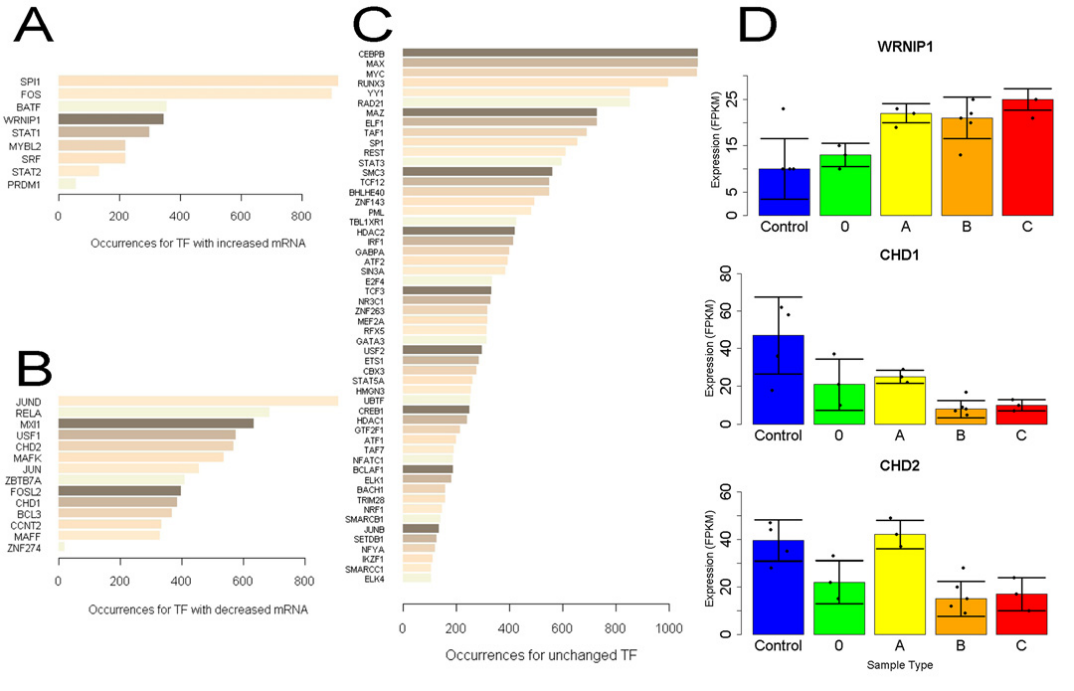
ENCODE ChIP analysis identifies factors which account for induced expression in SLE T cells. The number of chromatin immunoprecipitation binding sites within a 3kb window about significantly induced genes were counted **A)** Factors binding near induced genes which themselves are induced. **B)** Factors binding near these genes having reduced mRNA. **C)** ChIP factors with unchanged mRNA and more than 100 binding sites near induced loci. **D)** Expression of select chromatin factors by sample type, where error bars represent the median absolute deviation from the median.

We next looked at ENCODE immunoprecipitation data for factors detected in chromatin that might share responsibility for any observed expression changes. Examination of the body and 3kb flanking regions of genes induced at least 2-fold in SLE T cells revealed thousands of binding events. As the consortium data is derived from various cell lines, we looked at the expression of these binding ChIP targets in our data. Among those with signal at induced loci, nine had greater than 1.5-fold increased mRNA in SLE (Fig 7A), most of which are known to impact lymphocyte development or activity. Minimally characterized in T cells was WRNIP1 (Werner helicase interacting protein 1), an ATP-dependent DNA-binding protein related to DNA repair [29, 30]. As they show increased mRNA and are found at induced loci, these factors likely act as transcriptional activators.

A greater number of transcription/chromatin factors were reduced in expression (Fig 7B), a trend detected by DAVID analysis. Among the 14 ChIP targets with mRNAs reduced at least 1.5-fold, several have little described role in T cells. These include ZNF274, which recruits repressive factors SETDB1 and TRIM28/KAP1 [31], chromatin modifiers CHD1 and 2 [32] and ZBTB7A, which represses glycolytic genes [33].

Because mRNA levels are frequently unchanged for transcription factors directing an expression program, we checked ChIP targets unaltered in SLE and depict those with at least 100 sites at induced loci (Fig 7C). Several top hits are well known to influence T cell biology. Runx3 is critical for thymocyte development [34] and YY1 influences Th2 cytokine production [35]. SMC3 and RAD21 interact with MXI1 (found among ChIP targets with decreased mRNA) to function in the cohesin complex, and the former is associated with atopic asthma [36]. Expression for these ChIP factors was usually similar in Type B and C samples (Fig 7D).

## Conclusions

Patient variability challenges diagnosis and treatment of many diseases, and peripheral blood provides a window into health status capable of reporting on tissues throughout the body. The transcriptome of peripheral blood components show variation with circadian [37] and seasonal [38] periodicity and are growing in descriptive utility in autoimmunity [39] and other clinical settings including transplantation [40] heart failure [41] amyotrophic lateral sclerosis [42] and several cancers [43, 44] where patient subtypes can be identified based on tumor immune cell expression [45]. Expression analysis in disease-relevant tissue is also useful for prioritization of genomic variants [46].

SLE patients present great clinical heterogeneity as a result of genetic diversity and epigenetic changes related to immunological memory. Robust molecular diagnostics have the potential to guide treatment and describe the causes of the disease. Our mRNA analysis identified new genes related to T cell dysfunction and confirmed induction of interferon signature genes (ISG), including *OAS2* which we previously showed is specific to SLE autoimmunity [47]. Patient stratification by ISG expression, however, remains poorly correlated to disease activity [48]. While many of the pathways and domains we found are unsurprising, their notation will aid study of lymphocyte function. Induction of small groups of genes unique to single patients was unexpected, and may prove to be a source of pathological variability related to more common failures of transcriptional repression. Study of nuclear regulators may be most fruitful, in light of the persistent hypomethylation and expression activation displayed SLE T cells [49–51].

We expected patients with various clinical signs to show more distinctive expression patterns, as has been shown for rheumatoid arthritis [52]. Our data indicate that the extent of expression alteration in T cells correlates more with the severity of disease rather than which major symptoms were apparent. Both Type B and C sample groups had more genes with significant expression differences, and higher average SLEDAI scores, than did Type 0 in comparison to controls. We were encouraged that expression analysis detects subtypes of patients. Although these expression phenotypes do not correlate with specific symptoms, they will support patient stratification for study of SLE T cell function. We expect that patient subtypes, at least with regard to sexual dimorphism, will increase discriminatory power and reveal common symptoms or treatment response subsequent analysis of a larger cohort.

Several genes primarily associated with B cells, such as *CD38* and *MZB1*, had striking induction in SLE T cells. This may mark an aberrant or immature cell type that is resistant to negative selection T cell purification. Signals related to B cell activation may mistakenly be received on or within T cells, driving trans- or dedifferentiation to a close lineage member. Expression of immunoglobulin transcript fragments is perplexing, and their profound induction in some patients indicates a potential mechanism of disease, again, perhaps related to a transcriptional or chromatin regulatory lesion. While we saw no evidence of Ig protein, the number of loci and degree of induction for these and several genes with products located on the endoplasmic reticulum offer strong evidence that regulation of antibody production deserves further examination.

We conclude that study of diseases hampered by patient heterogeneity can be supported by high coverage transcriptomics of purified tissues, even in small cohorts. Unbiased detection of patient subtypes and biomarkers associated with symptoms may both be revealed if expression variability is carefully considered. The genes and domains suggested herein will hopefully aid in the study of SLE lymphocyte biology and eventually provide aid to clinical decision making.

## Supporting Information

S1 Data This file contains expression and comparison information extracted from S1 Table for use as input for analysis in R. (CSV)

S1 Fig T cell sample types B and C present the most alterations relative to controls. **A)** Gene counts for 1.5- and 2-fold expression changes (FPKM) apparent in various comparisons show that sample Types B and C have the most extreme expression phenotypes relative to controls. The number of samples used as input is listed in parentheses for each comparison. **B)** Overlaps of mRNAs increased or reduced at least 1.5-fold in abundance in three sets of three comparisons. Left, refinement effect on the overall control v SLE analysis. Middle, patient Types B and C show most of the altered genes found in Type 0 in addition to many others. Right, comparisons of clinical signs show greater similarity between increased dsDNA antibody and low complement samples, and that nephritis is accompanied by reductions in many mRNAs.
(TIF)

S1 Table This file contains the gene expression data resulting from the Cufflinks and Cuffdiff analysis, including FPKM and descriptions for the most annotated 24,263 human genes along with metrics from the comparisons performed. The raw sequence data has been deposited at the Sequence Read Archive under Bioproject Accession ID PRJNA293549.
(XLS)

S2 Table This file includes lists of ENSG IDs for all CUFFDIFF comparisons yielding greater than 1.5x mRNA expression changes and the David Analysis of Kegg pathway and Interpro domain terms for the refined SLE versus control comparison. (XLS)

S3 Table This file includes count-based expression data (matrix_counts) and the comparison matrix inputs for DESeq analysis and an overview of the results. (XLS)

S1 Text This document contains supplemental methods information including commands and scripts employed for informatics analysis. (DOC)

## Abbreviations

SLE: Systemic Lupus Erythematosus
PBMC: peripheral blood mononuclear cells
SLEDAI: Systemic Lupus Erythematosus Disease Activity Index
FPKM: Fragments Per Kilobase of transcript per Million mapped reads
FDR: False Discovery Rate
dsDNA: double-stranded DNA
