# Supplementary figures and images for: T Cell Transcriptomes Describe Patient Subtypes in Systemic Lupus Erythematosus
Source: PLoS One. 2015 Nov 6;10(11):e0141171. doi: 10.1371/journal.pone.0141171 (PMC4636226; doi:10.1371/journal.pone.0141171)

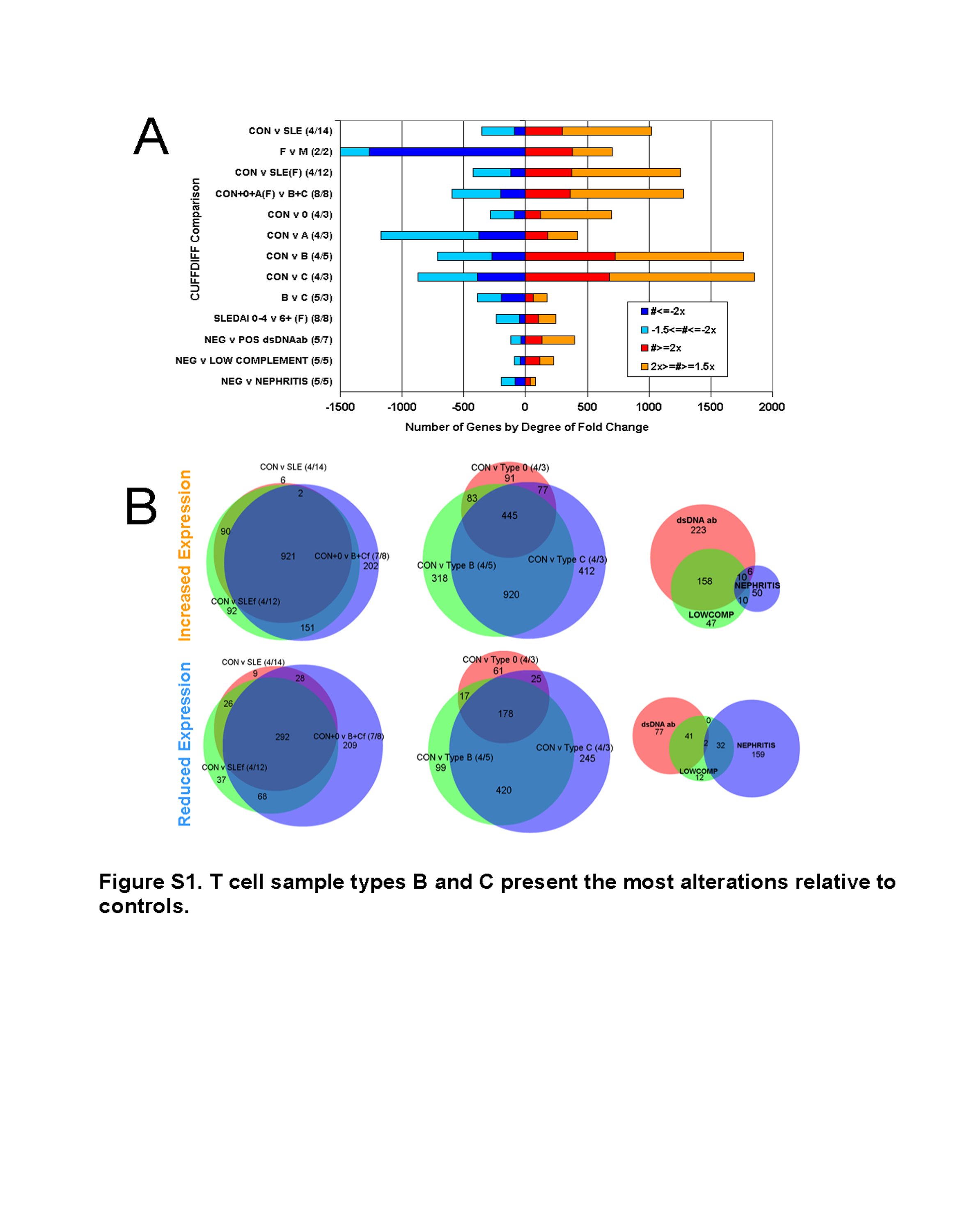

Supplement: S1 Fig — A) Gene counts for 1.5- and 2-fold expression changes (FPKM) apparent in various comparisons show that sample Types B and C have the most extreme expression phenotypes relative to controls. The number of samples used as input is listed in parentheses for each comparison. B) Overlaps of mRNAs increased or reduced at least 1.5-fold in abundance in three sets of three comparisons. Left, refinement effect on the overall control v SLE analysis. Middle, patient Types B and C show most of the altered genes found in Type 0 in addition to many others. Right, comparisons of clinical signs show greater similarity between increased dsDNA antibody and low complement samples, and that nephritis is accompanied by reductions in many mRNAs. (TIF) [file pone.0141171.s002.tif]
