# Supplementary material for: T Cell Transcriptomes Describe Patient Subtypes in Systemic Lupus Erythematosus
Source: PLoS One. 2015 Nov 6;10(11):e0141171. doi: 10.1371/journal.pone.0141171 (PMC4636226; doi:10.1371/journal.pone.0141171)
Supplement: S1 Text — (DOC) [file pone.0141171.s006.doc]

**Supplental Methods File for**

**“T cell transcriptomes describe patient subtypes in systemic lupus erythematosus”**

Sean J. Bradley^1^, Abel Suarez-Fueyo^1^, David R. Moss^2^, Vasileios C. Kyttaris^1^, George C. Tsokos^1^

1 Division of Rheumatology, Department of Medicine, Beth Israel Deaconess Medical Center, Harvard Medical School, Boston, Massachusetts, USA.

2 Department of Anesthesiology, Tufts Medical Center, Boston, Massachusetts, USA.

[sjbradle@bidmc.harvard.edu](mailto:sjbradle@bidmc.harvard.edu), [Bradley@Brandeis.edu](mailto:Bradley@Brandeis.edu) (S.J. Bradley), gtsokos@bidmc.harvard.edu (G.C. Tsokos).

**Sequencing and Analysis**

cDNA library preparation and sequencing was performed by BGI (Shenzhen, China). The libraries were paired end 80bp and unstranded. Around 75e6 read pairs were mapped per sample at roughly 85% mapping to HG19.

The data was assessed and manipulated using the following commands and programs in LSF syntax. In this format spaces and line breaks must be considered with care. We performed Tophat-Cufflinks analysis first and later DESeq2.

**FASTCQ for per-base read quality and over-represented reads**

bsub -N -q QUE -n 6 "/lab/software/FastQC/fastqc -t 20 -o ./fastqc ./data/L027_1.fq.gz"


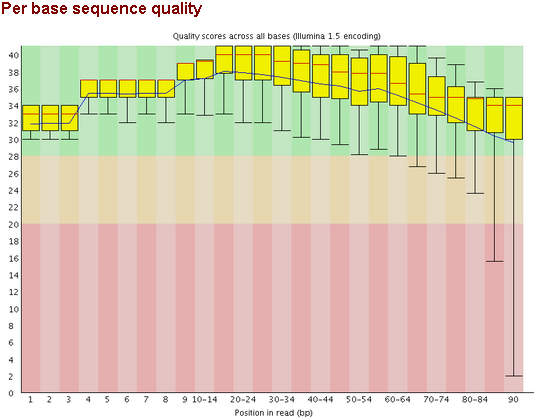

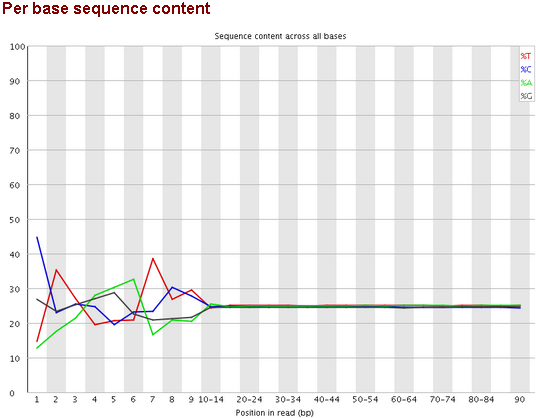


**SEQTQ trims poor quality ends of reads**

bsub –o outL027 -q QUE "/lab/software/seqtk/seqtk/seqtk trimfq -b 14 /path/L027.fastq > ./trimmed/L027t.fq"

**TOPHAT maps sequencing reads to the genome**

bsub -o outL027th -q QUE -R "rusage[mem=20]" "/lab/software/tophat-2.0.10.Linux_x86_64/tophat2 --num-threads 4 --bowtie1 --library-type fr-unstranded --output-dir ./tophat/L115 --GTF /lab/HsGenome/Gencode/v19/gencode.v19.chr_patch_hapl_scaff.annotation.gtf /lab/HsGenome/Gencode/v19/GRCh37.p13.genome ./trimmed/L027_1t.fq ./trimmed/L027_2t.fq"

**AlignmentMetricPickard provides QC on mapping**

bsub -o outL027aliMet -q QUE -R "rusage[mem=20]" "java -Xmx4024m -jar /lab/software/picard-tools-1.107/CollectAlignmentSummaryMetrics.jar TMP_DIR=/lab/sbradley/tmp/ VALIDATION_STRINGENCY=SILENT METRIC_ACCUMULATION_LEVEL=SAMPLE INPUT=./tophat/L027/accepted_hits.bam OUTPUT=./tophat/L027/Alignment_metrics.txt REFERENCE_SEQUENCE=/lab/HsGenome/Gencode/v19/GRCh37.p13.genome.fasta ASSUME_SORTED=false"

Left reads: Input : 76240074

Mapped : 73867771 (96.9% of input)

of these: 9199798 (12.5%) have multiple alignments (138633 have >20)

Right reads:Input : 76240074

Mapped : 73265859 (96.1% of input)

of these: 9108760 (12.4%) have multiple alignments (138646 have >20)

96.5% overall read mapping rate.

Aligned pairs: 71559851

of these: 8805894 (12.3%) have multiple alignments

564055 ( 0.8%) are discordant alignments 93.1% concordant pair alignment rate.

**RnaSEQmetricsPickard provides QC on mapping**

bsub -o outL027rnaSeqmetrics -q QUE -R "rusage[mem=8]" "java -Xmx4024m -jar /lab/software/picard-tools-1.107/CollectRnaSeqMetrics.jar TMP_DIR=/lab/sbradley/tmp/ VALIDATION_STRINGENCY=SILENT STRAND_SPECIFICITY=NONE REF_FLAT=/lab/pipeline/reference/rsem_ref_v19/refFlat.txt RIBOSOMAL_INTERVALS=/lab/pipeline/reference/rsem_ref_v19/gencode_v19.rRNA.interval_list INPUT=/lab/sbradley/tophat/L027/accepted_hits.bam OUTPUT=./tophat/L027/RNASeq_metrics.txt REFERENCE_SEQUENCE=/lab/HsGenome/Gencode/v19/GRCh37.p13.genome.fasta ASSUME_SORTED=false"

output:## METRICS CLASS net.sf.picard.analysis.RnaSeqMetrics

PF_BASES 1.12E+10, PF_ALIGNED_BASES 1.12E+10, RIBOSOMAL_BASES 9.44E+08

CODING_BASES 5.60E+09, UTR_BASES 2.67E+09, INTRONIC_BASES 4.77E+08

INTERGENIC_BASES 1.49E+09, IGNORED_READS 0, CORRECT_STRAND_READS 0

INCORRECT_STRAND_READS 0, PCT_RIBOSOMAL_BASES 0.084436, PCT_CODING_BASES 0.500651

PCT_UTR_BASES 0.239179, PCT_INTRONIC_BASES 0.042629, PCT_INTERGENIC_BASES 0.133107, PCT_MRNA_BASES 0.73983, PCT_USABLE_BASES 0.739787, PCT_CORRECT_STRAND_READS 0, MEDIAN_CV_COVERAGE 0.482153, MEDIAN_5PRIME_BIAS 0.129857, MEDIAN_3PRIME_BIAS 0.201806, MEDIAN_5PRIME_TO_3PRIME_BIAS 0.756247, SAMPLE , LIBRARY , READ_GROUP

**CUFFLINKS calculates expression for each sample**

bsub -o outL027cl -q QUE -R "rusage[mem=4000]" "/lab/software/cufflinks-2.1.1.Linux_x86_64/cufflinks -o ./cufflinks/L027/ --min-frags-per-transfrag 0 --GTF /lab/HsGenome/Gencode/v19/gencode.v19.chr_patch_hapl_scaff.annotation.gtf ./tophat/L027/accepted_hits.bam"

These per-sample expression values were used to build the mastersheet.

**CUFFDIFF calculates expression difference in a comparison**

NOTE: CHANGE LOGNAME AND OUTPUT DIRECTORY, RUN IN TOPHAT FOLDER, INSERT SAMPLENAMES BEFORE LAST QUOTE.

bsub -o outCDDSabWTvNEG -q QUE -R "rusage[mem=20]" "/lab/software/cufflinks-2.1.1.Linux_x86_64/cuffdiff -o ./cuffDiffWTvNEGdna/ --min-alignment-count 0 /lab/HsGenome/Gencode/v19/gencode.v19.chr_patch_hapl_scaff.annotation.gtf ./N048/accepted_hits.bam,./N068/accepted_hits.bam,./N090/accepted_hits.bam,./N098/accepted_hits.bam ./L102/accepted_hits.bam,./L072/accepted_hits.bam,./L062/accepted_hits.bam,./L005/accepted_hits.bam,./L101/accepted_hits.bam"

We took columns status, value_1, value_2, log2(fold_change) and q_value from the gene_exp.diff file for each comparison to build the master sheet.

**MasterSheet Preparation**

Most data handling occurred in MS Excel, where it is advisable to save multiple forms for different uses. The first form includes all “gene” entries resulting from the TOPHAT pipeline, which are dependent on the GTF used for mapping. This sheet includes Gene symbol and ENSG identifiers, both of which have advantages in different analyses. Next we placed CUFFLINKS per sample expression data, several columns of descriptive information for each gene, followed by the results of many CUFFDIFF comparisons.

We then discarded entries with poor gene descriptions or lacking standard gene symbols, including many pseudogenes and noncoding RNAs, leaving the 24263 best annotated genes. These were then stratified by average expression of all samples into high, medium, low and unexpressed classes. Most analysis was performed on genes in the high and medium classes, which include the top quartile of expression in the genome, with average FPKM values of 11 or greater. Digits to the right of the “.” In the ENSG identifiers were removed to make them compatible with various databases.

The expression data was converted to numeric and values less than 1 were converted to a pseudocount of 0.1 FPKM to avoid arithmetic failures downstream. CUFFLINKS failed in some cases to register reads although they were visible in IVG, perhaps as a result of excessive read mapping or tissue specific isoforms insufficiently recognized by in the GTF. Some cases are more obscure, such as for PTPN6, which was found to be expressed in CUFFDIFF and not CUFFLINKS. The “modified mean” calculated by CUFFDIFF was routinely lower than that calculated manually using the CUFFLINKS expression values for a sample group.

**Post Analysis Scripts in R.**

Code should be copied to a text editor for examination to avoid line wrapping issues. These were implemented in R Studio version 0.98.1091 with R 3.1.3 and the working directory is set to the script location, usually ~/Downloads, and several support files are in folder ~/Downloads/TcellTF.

**Tcell_MannWhitney.R**

# Applies Mann-Whitney nonparametric test to compare two groups of expression values

# export plot 800x400 plot window much be large

# set threshold at p.value, default is 0.01

rm(list = ls(all = TRUE)) # Clear Workspace

par(mfrow=c(1,1))

file <- read.csv('./TCellTF/GED_AB.csv', colClasses = "character")

file <- file[1:6094,]

#DSabNEGvPOS

#genesB <- as.numeric(file[x,c('L102.1','L072.1','L062.1','L005.1','L101.1')])

#genesC <- as.numeric(file[x,c('L078.1','L074.1','L133.1','L137.1','L115.1','L027.1','L031.1')])

#REGvLOCOMP

#genesB <- as.numeric(file[x,c('L101.1','L078.1','L102.1','L027.1','L005.1','L062.1')])

#genesC <- as.numeric(file[x,c('L115.1','L133.1','L074.1','L031.1','L072.1','L137.1')])

#nephritis

#genesB <- as.numeric(file[x,c('L005.1','L101.1','L062.1','L031.1','L133.1','L027.1','L078.1')])

#genesC <- as.numeric(file[x,c('L137.1','L072.1','L074.1','L102.1','L115.1')])

mw_logical <- c()

for (i in file$Symbol) {

x <- which(file$Symbol == i)

genesALL <- as.numeric(file[x,30:47])

genesA <- as.numeric(file[x,30:33])

#DSabNEGvPOS

genesB <- as.numeric(file[x,c('L102.1','L072.1','L062.1','L005.1','L101.1')])

genesC <- as.numeric(file[x,c('L078.1','L074.1','L133.1','L137.1','L115.1','L027.1','L031.1')])

genesDF <- data.frame(

Expression =c(genesA, genesB, genesC),

Group =factor(rep(c("Control", "Neg", "Pos"),

times=c(length(genesA), length(genesB), length(genesC))))

)

# change P value below

result <- wilcox.test(genesB,genesC, correct=FALSE)$p.value <0.01

mw_logical <- c(mw_logical, result)

}

mwTRUE <- file$Symbol[which(mw_logical == TRUE)] ## Genes passing threshold

mwTRUE # will list the genes in the console

**Tcell_simplePlot.R creates plots such as in Figure 2.**

# export plot 700x400 plot window much be large

# plotting medians was more useful but error bars as stdev or MAD is debatable

# getwd() # set to source file

# setwd("~/Downloads")

rm(list = ls(all = TRUE)) # Clear Workspace

par(mfrow=c(1,1))

file <- read.csv('./TcellTF/GED_AB.csv', colClasses = "character")

file <- file[1:6094,]

# names(file) #will list column headers

# the following group definitions are to be used one at a time in the geneplot function

## CONvSLEf

#genesB <- as.numeric(file[x,c('N090.1','N068.1','N098.1','N048.1')])

#genesC <- as.numeric(file[x,c('L005.1','L101.1','L062.1','L031.1','L133.1','L027.1','L078.1','L137.1','L072.1','L074.1','L102.1','L115.1')])

## REGvLOCOMP

#genesB <- as.numeric(file[x,c('L101.1','L078.1','L102.1','L027.1','L005.1','L062.1')])

#genesC <- as.numeric(file[x,c('L115.1','L133.1','L074.1','L031.1','L072.1','L137.1')])

## DSabNEGvPOS

#genesB <- as.numeric(file[x,c('L102.1','L072.1','L062.1','L005.1','L101.1')])

#genesC <- as.numeric(file[x,c('L078.1','L074.1','L133.1','L137.1','L115.1','L027.1','L031.1')])

## nephritis

#genesB <- as.numeric(file[x,c('L005.1','L101.1','L062.1','L031.1','L133.1','L027.1')])

#genesC <- as.numeric(file[x,c('L137.1','L072.1','L074.1','L102.1','L115.1')])

## prednisone

#genesB <- as.numeric(file[x,c('L005.1','L101.1','L062.1','L078.1')])

#genesC <- as.numeric(file[x,c('L137.1','L072.1','L074.1','L102.1','L133.1')])

geneplot <- function (gene) {

x <- which(file$Symbol == gene)

genesALL <- as.numeric(file[x,30:47])

genesA <- as.numeric(file[x,30:33])

### REPLACE GROUP DEFINITION BELOW AND UNCOMMENT TWO GENES LINES

# nephritis

genesB <- as.numeric(file[x,c('L005.1','L101.1','L062.1','L031.1','L133.1','L027.1')])

genesC <- as.numeric(file[x,c('L137.1','L072.1','L074.1','L102.1','L115.1')])

###

medians <- sapply(list(genesA, genesB, genesC), median)

stdevs <- sapply(list(genesA, genesB, genesC), sd)

mads <- sapply(list(genesA, genesB, genesC), mad)

names(medians) <- c("Control", "Negative", "Positive") #,"All"

par(cex.lab=1.5)

plotMax <- 1.03*max(genesA, genesB, genesC)

bp <- barplot(medians, ylim=c(0,plotMax), ylab = "Expression (FPKM)",

cex.axis = 2, col=c("blue","cyan","purple"),

xlab= "Sample Type",

main = gene,cex.main=2,cex.names=2,

)

arrows(bp, medians-mads, bp, medians + mads, bp, angle = 90, code = 3, lwd=3)

#arrows(bp, medians-stdevs, bp, medians + stdevs, bp, angle = 90, code = 3, lwd=3)

points(jitter(rep(bp[1,1],length(genesA)),amount=0.2),genesA, pch = 21, bg = "black")

points(jitter(rep(bp[2,1],length(genesB)),amount=0.2),genesB, pch = 21, bg = "black")

points(jitter(rep(bp[3,1],length(genesC)),amount=0.2),genesC, pch = 21, bg = "black")

}

geneplot("C1orf86")

**Tcell_deltaLogList.R**

## makes multilist of ENSG ID using delta (max-min) log2(A/C) filtering

## set thresholds in sub2 for filter level BOTH OF THEM operator and number

## use names(file) to get column header names

## creates file in which must be renamed for each filtering.

rm(list = ls(all = TRUE)) # Clear Workspace

file <- read.csv('./TcellTF/GED_AB.csv', colClasses = "character")

run_names <- names(file)[seq(62,200, by=6)] # each comp has 6 data col, starting at 62

#Iterate filter and generate a list for each run.

ENSGlist <- list()

for (i in seq(62,200, by=6)) { # each comp has 6 data col, starting at 62

delta <- abs(as.numeric(file[,i+1]) - as.numeric(file[,i+2]))

#

# Set filtering threshold, default is delta of 5 and 1.5x fold change (=log2 0.6)

#

sub2 <- subset(file, delta >= 5 & as.numeric(file[,i+3]) >= 0.6)

#

# then repeat for <=-0.6 after renaming output file

# sub2 <- subset(file, delta >= 5 & as.numeric(file[,i+3]) <=-0.6)

#

output <- sub2[,24] #get column 24 ENSG IDs for DAVID analysis.

ENSGlist[[(i-56)/6]] <- output

}

#Filter using Q value in addition to delta and log2 cutoffs:

#for (i in seq(45,213, by=6)) { # here i is the name/ok column

# delta <- abs(as.numeric(file[,i+1]) - as.numeric(file[,i+2]))

# sub2 <- subset(file, delta >= 5 & as.numeric(file[,i+3]) <= -0.6 & as.numeric(file[,i+4]) <= 0.05)

# output <- sub2[,24] #get column 24 ENSG IDs for DAVID analysis.

# ENSGlist[[(i-56)/6]] <- output

#}

names(ENSGlist) <- run_names

sapply(ENSGlist, length) #tells you how many genes are in each category

ENSGlist[["CONAvBC_F"]] #chooses list element for specific run to check

#output csv, normally cant have columns of different length

max <- max(sapply(ENSGlist, length))

ENSGdf <- data.frame(matrix(NA, nrow = max, ncol = 24))

for (i in 1:24) {

ENSGdf[1:length(ENSGlist[[i]]),i] <- ENSGlist[[i]]

}

names(ENSGdf) <- run_names

write.csv(ENSGdf, "FilteredENSG_perCOMP_RENAME.csv", row.names=FALSE, na='')

**BedHeader.txt**

## MAKE BED FOR HUMAN TF BINDING, place this in /TcellTF

## browser lines log2 on TOTAL WT v SLE

browser position chr3:32992232-32997238

browser pix1120

browser hide all

#browser dense

#browser pack

#browser squish mrna

#browser full

##name=<track_label> - Defines the track label that will be displayed to the left of the track in the Genome Browser window, and also the label of the track control at the bottom. up to 15 characters, and enclosed in quotes if the text contains spaces. We recommend that the track_label be restricted to alpha-numeric characters and spaces to avoid potential parsing problems. The default value is "User Track".

##description=<center_label> - Defines the center label of the track in the Genome Browser window. The description can consist of up to 60 characters, and must be enclosed in quotes if the text contains spaces. The default value is "User Supplied Track".

##visibility=<display_mode> - Defines the initial display mode of the annotation track. Values for display_mode include: 0 - hide, 1 - dense, 2 - full, 3 - pack, and 4 - squish. The numerical values or the words can be used, i.e. full mode may be specified by "2" or "full". The default is "1".

##color=<RRR,GGG,BBB> - Defines the main color for the annotation track. The track color consists of three comma-separated RGB values from 0-255. The default value is 0,0,0 (black).

##itemRgb=On - If this attribute is present and is set to "On", the Genome Browser will use the RGB value shown in the itemRgb field in each data line of the associated BED track to determine the display color of the data on that line.

##useScore=<use_score> - If this attribute is present and is set to 1, the score field in each of the track's data lines will be used to determine the level of shading in which the data is displayed. The track will display in shades of gray unless the color attribute is set to 100,50,0 (shades of brown) or 0,60,120 (shades of blue). The default setting for useScore is "0".

##group=<group> - Defines the annotation track group in which the custom track will display in the Genome Browser window. By default, group is set to "user", which causes custom tracks to display at the top of the window.

##priority=<priority> - When the group attribute is set, defines the display position of the track relative to other tracks within the same group in the Genome Browser window. If group is not set, the priority attribute defines the track's order relative to other custom tracks displayed in the default group, "user".

##db=<UCSC_assembly_name> - When set, indicates the specific genome assembly for which the annotation data is intended; the custom track manager will display an error if a user attempts to load the track onto a different assembly. Any valid UCSC assembly ID may be used (eg. hg18, mm8, felCat1, etc.). The default setting is blank, allowing the custom track to be displayed on any assembly.

##offset=<offset> - Defines a number to be added to all coordinates in the annotation track. The default is "0".

##url=<external_url> - Defines a URL for an external link associated with this track. This URL will be used in the details page for the track. Any '$$' in this string this will be substituted with the item name. There is no default for this attribute.

##htmlUrl=<external_url> - Defines a URL for an HTML description page to be displayed with this track. There is no default for this attribute. A template for a standard format HTML track description is here.

##chr16 67877634 67909470 NUTF2 0.7

track name="defaultName" description="defaultBEDdesc" color=0,40,0 visibility=4 priority=1 useScore=1

**Tcell_TFmakeBedfile.R**

# searches for qualified gene rows in GED_wAB.csv

# note the complete mastersheet has changes relative to this csv file

# makes bedfiles with flanking 3kb regions of those genes

# renameNewUP2x.bed and renameNewDN2x.bed

# use to intersect with TXNchipV1 at UCSC

# files will be appended if not renamed

# setwd("~/Downloads/")

rm(list = ls(all = TRUE)) ## Clear Workspace

file <- read.csv('./TcellTF/GED_AB.csv', colClasses = "character")

text <- readLines('./TcellTF/BedHeader.txt')

# names(file) to find column headers

# default is to use log2.5 column for the CONAvBC_F Comparison

writeLines(text, "./TcellTF/renameNewUP2x.bed")

write.table(subset(file, log2.5 >= 1, select=206:210), #for induction greater than 2x...

"./TcellTF/renameNewUP2x.bed", #get coordinates starting col 206

quote = FALSE, #column number should be verified

row.names = FALSE, #if multiple input tables

col.names = FALSE, #are being used.

sep = "\t",

append=TRUE)

writeLines(text, "./TcellTF/renameNewDN2x.bed") #for 2x decrease in that comparison...

write.table(subset(file, log2.5 <= -1, select=206:210),

"./TcellTF/renameNewDN2x.bed",

quote = FALSE,

row.names = FALSE,

col.names = FALSE,

sep = "\t",

append=TRUE)

# names(bed) <- c("chr", "start", "end", "id", "log2")

Create login for UCSC to save tracks. Upload custom track ~/TcellTF/renameNewUP2x.bed and go to table browser.

Set region to genome, set intersection to "Regulation" and "Txn Factor ChIP" and "Txn Factor ChIP(wg..V3), set to all user tracks that have any overlap and submit. Set output format to GTF Genome Transfer Format and then click "Get Output" the text of which should be copied to a .txt, not saved as .txt.


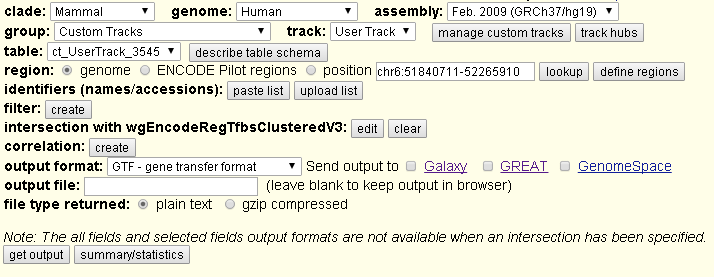
(UCSC image)

This contains factors detected by ChIP in the vicinity of the genes with 2x increased expression in SLE. Save as ~/TcellTF/tableA.txt and use the next script to plot the frequency of each ChIP factor.

**Tcell_TFgeneFreqTables.R**

## GeneFreqTable takes gtf output of intersection of chip with flanking regions

## gets exp data for the TFs from GED10_w

## makes 3 plots

## may have to resize plot window in rstudio for size fail

rm(list = ls(all = TRUE)) ## Clear Workspace

tx <- read.table('./TcellTF/tableA', stringsAsFactors = FALSE)

genes <- tx[,10] #get gene names from GTF table dl from UCSC

genesUnq <- unique(genes)

genesUnqDF <- data.frame("Symbol" = genesUnq)

file <- read.csv("TcellTF/GED_AB.csv", colClasses = "character") #exp data

file_genesUnq <- merge(genesUnqDF, file, by.x = "Symbol")

gene_simple <- file_genesUnq[,c(1,57,69:72)] #only col for name, i and CONvSLEf expression

genes.list <- table(genes)

genes.listdf <- as.data.frame(genes.list)

names(genes.listdf) <- c("Symbol", "Occurrences")

gene_simple2 <- merge(genes.listdf, gene_simple, by.x = "Symbol")

## why two merges

delta <- abs(as.numeric(gene_simple2$V1.1) - as.numeric(gene_simple2$V2.1))

sub2 <- subset(gene_simple2, delta >= 5 & as.numeric(gene_simple2$log2.1) >= 0.6)

sub3 <- subset(gene_simple2, delta >= 5 & as.numeric(gene_simple2$log2.1) <= -0.6)

sub4 <- subset(gene_simple2, as.numeric(gene_simple2$V1.1) >= 11 & as.numeric(gene_simple2$Occurrences) > 100 & as.numeric(gene_simple2$Occurrences) < 1300 & as.numeric(gene_simple2$log2.1) < 0.6 & as.numeric(gene_simple2$log2.1) > -0.6)

#sub5 <- subset(gene_simple2, interest == "i")

par(mfrow=c(1,1)) #was 3,1

## UNCOMMENT THESE THREE PLOTS ONE AT A TIME

## Rstudio Plots Window must be large enough to avoid errors

# export png 500x350

#barplot(sub2$Occurrences[order(sub2$Occurrences)],

# names = sub2$Symbol[order(sub2$Occurrences)],

# horiz = TRUE,

# las = 1, # las gives orientation of axis labels

# col = c("beige", "blanchedalmond", "bisque1", "bisque2", "bisque3", "bisque4"),

# border = NA, # No borders on bars

# cex.names = 0.8,

# xlab = "Occurrences for TF with increased mRNA")

# export png 500x400

#barplot(sub3$Occurrences[order(sub3$Occurrences)],

# names = sub3$Symbol[order(sub3$Occurrences)],

# horiz = TRUE,

# las = 1, # las gives orientation of axis labels

# col = c("beige", "blanchedalmond", "bisque1", "bisque2", "bisque3", "bisque4"),

# border = NA, # No borders on bars

# cex.names = 0.8,

# xlab = "Occurrences for TF with decreased mRNA")

# export png 500x800

barplot(sub4$Occurrences[order(sub4$Occurrences)],

names = sub4$Symbol[order(sub4$Occurrences)],

horiz = TRUE,

las = 1, # las gives orientation of axis labels

col = c("beige", "blanchedalmond", "bisque1", "bisque2", "bisque3", "bisque4"),

border = NA, # No borders on bars

cex.names = 0.7,

xlab = "Occurrences for unchanged TF")

**Tcell_medNormForGCT.R**

This script was used initially for gene groups of interest and then simply applied to the whole transcriptome for ease of use.

# normalizes data about the median and scale difference

# from the median by the median absolute deviation (MAD),

# or mean abosolute deviation. GCT format is used in Gene-e

# and Genepattern.

#

# input file is medNormInput.gct in gct format, which has two lines:

# #1.2 ##format ID

# 6094 18 ##number of data rows, number of samples

# for this script there are two gene IDs in the leftmost two columns

# output is medNormOut.gct

# setwd("~/Downloads")

file <- read.table('medNormInput.gct', sep = "\t", skip = 2, header = TRUE)

data <- file[,3:ncol(file)]

genelist <- file[,1:2]

gct_zscore <- cbind(genelist, round(t(scale(t(data))), digits=3))

gct_rzscore <- cbind(genelist, round(t(scale(t(data), center = apply(data,1,median), scale = apply(data,1,mad))), digits=3))

line1<- "#1.2"

line2<- dim(data)

gct <- function(name="medNormOut.gct", method="median") {

write(line1, name)

write(line2, name, sep = "\t", append=TRUE)

if (method == "mean") { output <- gct_zscore }

else if (method == "median") { output <- gct_rzscore }

else { return ("no such method") }

write.table(output, name,

quote = FALSE,

row.names = FALSE,

col.names = TRUE,

sep = "\t",

append=TRUE)

}

gct ()

**DEseq2 Pipeline: Sort accepted hits, Htseq Count, DEseq analyze**

Sort Tophat Accepted hits bam files by name with samtools:

bsub -o N048.out -q short -W 3:00 "samtools sort -n -o N048_hits.bam N048_hits_temp > N048_hits.sort.bam"

1.5hr each on 10g input 15g result. Keep space quota in mind silent failures may result if reached without temp scratch space.

du --max-depth 1

Count reads per gene. Renamed GTF to gencode.v19.sjb01.gtf

module load seq/htseq/0.6.1p1

using loop:

for i in *sort.bam; do bsub -q short -W 7:00 -R "rusage[mem=60000]" "htseq-count --order=name -f bam --stranded no $i gencode.v19.sjb01.gtf > $i.count.txt"; done

per file: (example CPU time: (~6hrs) Max Memory: 171MB Max Swap: 539 MB Max Processes: 3 Max Threads: 4)

bsub -q short -W 7:00 -R "rusage[mem=60000]" "htseq-count --order=name -f bam --stranded no L115_hits.sort.bam gencode.v19.sjb01.gtf > L115_hits.sort.bam.count.txt"

Failures will occur for imperfect name sorting. Last line in successful outfile: 113654681 SAM alignment pairs processed.

Preprocess results for Deseq. Moved counts to ~/tophat/Bams/htcounts

module load stats/R/3.1.2

# change directory to a folder with ONLY .txt files in it

cd ~/tophat/Bams/htcounts

# start R

R

#setwd for htseq-count output files. only these .txt files can be in this directory

setwd("~/tophat/Bams/htcounts")

fileName <- dir()

#read in 1 file and view headers

fileone<-read.table(fileName[1], header=F, sep="\t")

head(fileone)

#initiate rawcounts

rawcounts <- NULL

rawcounts <- data.frame

#loop through directory (have no additional files in directory). Pull column 2, the "rawcounts", append to rawcount

#copy from "for" to "dim"

for (i in 1:length(fileName)){

file <- read.table(fileName[i], header=F, sep="\t")

rawcounts <- cbind(rawcounts, file[,2])

}

dim(rawcounts) #[1] 63573 19

head(rawcounts)

#get the annotation names from the first column of 1 file

names<-fileone[1][,1]

names<-as.data.frame(names)

#turn from factor into character (was read in as factor because some samples start with numbers)

namesc<-apply(names, 2, as.character)

#create counts_matrix binding column 1 from namesc to the rawcounts matrix (minus the ? column)

counts_matrix<-NULL

counts_matrix<-cbind(namesc[,1], rawcounts[,-1])

head(counts_matrix)

#not recording output here

#writes the counts matrix to the directory above the text files, keeping the text files directory clean

write.table(counts_matrix, file="../counts_matrix.txt", sep="\t")

#txt appears in upper directory.

Run Deseq2 in R

Output includes results tables at 4 different FDR levels, as well as smear, MDS, and BCV-like (dispersion vs. count number) plots. Set the directory to folder with matrix of comparisons and matrix of counts. Verify filenames referenced in the script. Run locally took ~10min.

Change run number in 4 places: three plot names in the "plots" function, and in the lapply loop at the bottom of the "write_results" function.

**Tcell_DEseq2_process.R**

#DESeq2 analysis, inputs: matrix_comparisons.txt matrix_counts.txt

#Westerman, Kenneth Edwards

#Holton, Kristina Marie

#Bradley, Sean Joseph (Bradley@Brandeis.edu)

#matrix_counts.txt remade often, needs nonredundant 5 lines at bottom

########## REQUIRED PACKAGES ##########

if (!require("DESeq2")) { #Differential expression analysis

source("http://bioconductor.org/biocLite.R")

biocLite("DESeq2")

library(DESeq2)

}

if (!require("ggplot2")) { #Plots

install.packages("RColorBrewer", dependencies = TRUE)

library(RColorBrewer)

}

if (!require("gplots")) { #Plots (heat map)

install.packages("gplots", dependencies = TRUE)

library(gplots)

}

if (!require("ggplot2")) { #Plots (heat map)

install.packages("ggplot2", dependencies = TRUE)

library(ggplot2)

}

########## DATA IMPORT ##########

rm(list = ls(all = TRUE)) ## Clear Workspace

setwd("~/Downloads/TcellTF") #script, matrix_counts, matrix_comparison location

countData0 <- read.delim("matrix_counts.txt", row.names=2) #Load counts matrix, genes as row names

countData <- countData0[,-1] #removed index col1 "Remove redundant genes column"

countData <- head(countData,-5) #Remove unwanted last 5 rows (no_feature, alignment_not_unique, etc.)

#colData contains metadata about each sample, namely the appropriate labels for each of the comparisons

colData <- read.delim('matrix_comparisons.txt')

rownames(colData) <- colData[,1]

colData <- colData[,-1]

########## FUNCTION DEFINITIONS ##########

#Filter for comparison-relevant samples, format data as a DESeq dataset

formatting <- function (countData, colData, comparison) {

notNA <- !is.na(colData[,comparison]) #Find relevant samples for the comparison

countData <- countData[,notNA] #Subset countData to only relevant samples (columns)

colData <- colData[notNA,] #Subset colData to only relevant samples (rows)

colData[,comparison] <- relevel(colData[,comparison], "no") #Choose "no" as baseline factor level

dds <- DESeqDataSetFromMatrix(countData = countData, #Format data as a DESeq dataset

colData = colData,

design = as.formula(paste("~", comparison))) #Only covariate is the current comparison

return(dds)

}

#Append counts matrix to results table and write to file for a series of FDR thresholds

write_results <- function(res, comparison) {

res_withCounts <- merge(data.frame(res), countData, by=0) #Append count data to results

rownames(res_withCounts) <- res_withCounts[,1] #Genes become row names

res_withCounts <- res_withCounts[,-1] #Delete redundant gene column

#Function tests a vector for values under a threshold (need user-defined function to deal with NA values)

meets_threshold <- function(value, threshold) {

response <- value < threshold #TRUE if value < threshold, FALSE otherwise, NA if value == NA

response[is.na(response)] <- FALSE #Rows w/ NA become false (meaning they will be discarded)

return(response) #Return a vector of T/F indicating whether the threshold is met

}

#Function filters data frame for columns with padj < threshold

filtered <- function(df, threshold) {

select <- meets_threshold(df$padj, threshold)

return(df[select,])

}

fdr_levels <- c(0.05, 0.01, 0.001, 0.0001) #List FDR thresholds of interest

#For each FDR, create a table with that padj threshold -- "output" is a list of data frames w/ results and counts

results_list <- lapply(fdr_levels, function(x) filtered(res_withCounts, x))

names(results_list) <- c('0.05','0.01','0.001','0.0001') #Name each data frame in "output" according to FDR threshold

#Write results at each threshold to tab-separated files

lapply(names(results_list), function(x) write.table(results_list[[x]], paste0(comparison,'_results_', x, '.run1.txt'), sep='\t', quote=F))

}

#Create smear, MDS, and BCV-like plots

plots <- function(dataset, res, log_counts, comparison) {

#Smear plot: shrunken log fold changes vs. mean expression

png(paste0(comparison,'_smear_plot.run1.png'))

plotMA(res, main="DESeq2 Results", ylim=c(-2,2))

dev.off()

#MDS Plot: sample similarity comparison through dimensionality reduction (a.k.a. PCoA)

distMatrix <- as.matrix(dist(t(log_counts))) #Compute distance (dissimilarity) matrix

mds <- data.frame(cmdscale(distMatrix)) #Multidimensional scaling on distance matrix

mds <- cbind(mds, as.data.frame(colData(dataset))) #Append sample metadata to MDS coordinates

png(paste0(comparison,'_MDS_plot.run1.png'))

print(qplot(X1, X2, color=get(comparison), data=mds, main="MDS Plot")) #Create MDS plot color-coded by condition

dev.off()

#Plot of dispersion (original and shrunken estimates) vs. mean expression level

png(paste0(comparison, '_BCV-like_plot.run1.png'))

plotDispEsts(dataset)

dev.off()

}

#Run a single comparison through formatting, analysis, and plot creation

pipeline <- function(countData, colData, comparison) {

dds <- formatting(countData, colData, comparison) #Filter for comparison-relevant samples, format data as a DESeq dataset

dds <- DESeq(dds) #Run DESeq differential expression analysis on dataset

#Steps: estimate size factors, estimate dispersions, negative binomial Wald test

res <- results(dds) #Store results of analysis

write_results(res, comparison) #Append counts matrix to results table and write to file for a series of FDR thresholds

log_counts <- log2(counts(dds, normalized=T) + 1) #Shifted log transform = log2(n+1)

plots(dds, res, log_counts, comparison) #Create smear, MDS, and BCV-like plots

}

########## ONE LINE TO RUN THEM ALL, ONE LINE TO FIND THEM ##########

lapply(colnames(colData), function(x) pipeline(countData, colData, x))

**GENE-E Clustering**

We used version 3.0.242 Dev (Built 03/09/2015) downloaded from the Broad Institute. This program uses .gct files but can work with simple flat files. Perhaps load by File>Open>medNormOut.gct output of Tcell_medNormForGCT.R but more frequently we generated non-gct files by filtering the masterlist, following application of the medNorm script to expression for all genes. For instance genes with a 1.5-fold change (custom conditional filter including greater than or equal to 0.6 AND less than or equal to -0.6) in a comparison could be obtained by selection in Excel of the leftmost 23 columns (gene names, descriptors and data for 18 samples) and top 100 rows including the header, and pasting to a .txt file. Some header names, such as description, can disrupt this approach. After loading the file Tools>Clustering> >Hierarchical Clustering (or the dendrogram icon) can be applied, where we routinely clustered columns and rows with average linkage one minus Pearson correlation. We found the data most informative after View>Preferences options color scheme tab was set to

Relative and the Relative Setting as “Subtract row median divide by row median absolute deviation” although different approaches have different strengths. To test these settings select row and column IDs and apply Visualization>Row Profile Plot (or similar icon) to test how cell coloration represents what relative expression differences. These sample and gene description IDs can be copied to text for other purposes by right clicking. The dendrogram coloration can be altered by dragging the dotted line at the top or left of the image. File>Save Image as PDF was the output usually employed.

**NMF clustering at Genepattern server**

Provided by the Broad Institute.

http://genepattern.broadinstitute.org/gp/pages/index.jsf

Expression data was first run through the PreprocessDataset module created by Joshua Gould ([gp-help@broadinstitute.org](mailto:gp-help@broadinstitute.org)) which requires strict adherence to .gct format (including two description columns, only numeric data values).

Input filename GED_AB.gct, threshold and filter: yes, floor threshold: 1, ceiling: 20000, min fold change: 2, min delta: 5 (max-min), num outliers to exclude: 1, row normalization: no, row sampling rate: 1 (use all), threshold for removing rows (10min FPKM), number of columns above threshold (10 samples must >10), log2 transform: yes, output file format: same

On the files tab, select the output and save it locally, and also save it to the uploads folder to apply to NMF clustering.

Non-negative Matrix Factorization Consensus Clustering (Pablo Tamayo Jean-Philippe Brunet and Ted Liefeld). Run with defaults initially then increase number of clusterings to confirm. Input dataset: gct, k initial* 2, k final* 20, num clusterings* 20 then 100, max num iterations* 2000, error function* divergence, random seed* 123456789, stop convergence* 40, stop frequency* 10.

Similar results were obtained with different numbers of input genes but the figure was derived from consensus.plot.K4.pdf created by using class A+B (6094 top expressed genes, 3820 after preprocessing) which was most consistent with the Gene-e Pearson result.

**SVD implementation at http://biographserv.com/**

This tool first normalizes gene counts to reads per million, and then transforms the expression to natural log values. Next low expression genes are removed, where no sample is greater than log 3 (therefore requiring at least one of the samples to have reads being >= 0.1% of total reads in that sample). Then the Numpy Linear Algebra library function SVD is applied (see [http://docs.scipy.org/doc/scipy/reference/generated/scipy.linalg.svd.html](https://email.caregroup.org/owa/redir.aspx?C=lGvSVJO5-02Nk5sHd6Q5cx_5_xaVNNJIeNJJSgDrp6i0yU85XP2q7S4lmPujHHtE4FBz_WcxWa8.&URL=http%3A%2F%2Fdocs.scipy.org%2Fdoc%2Fscipy%2Freference%2Fgenerated%2Fscipy.linalg.svd.html)). Then a scree plot is produced indicating how much of the total variance is attributed to each dimension, where the 2D plot shows eigenvector 1 vs 2. We used class A genes (top 2400 expressed) as input, and the figure was enhanced by increasing the font size and adding colored circles in PowerPoint.

**DAVID Analysis of KEGG Pathways and Interpro Domains**

<http://david.abcc.ncifcrf.gov/summary.jsp> Lists of differentially expressed genes in each CUFFDIFF comparison were generated with Tcell_deltaLogList.R. On the upload tab at left under Step 1 paste a list of ENSG IDs, then set the identifier to ENSEMBLE_GENE_ID, set as gene list and then submit. The use of different background lists, for instance only the “expressed” genes in a sample, had no more than a 2-fold improvement in P value and so was skipped for simplicity and to make the approach conservative. Within the many Annotation Summary Results, we used Pathways>Kegg Pathways>Chart output and copied the contents to Excel for record keeping and sorting. During a session, clicking on the blue bar will produce the relevant list of genes, as long as only one pathway window is open. This list of genes was also copied to Excel to maintain access to hyperlinks, and DAVID provides the best gene summaries available in GENERIF_SUMMARY with direct literature links. We used KEGG and INTERPRO because they were so efficient but many other ontology types are very informative.

**Tcell_KWDunn.R**

This script was used to find genes whose expression was most different in different comparisons. It permits comparison between multiple groups more easily than CUFFDIFF and DESeq work on 2 groups. Some would argue that use of this statistic is improper given the number of tests being employed, but it is the best manner of prioritization we found to find genes marking the patient subtypes. Asking which genes are unique to a subgroup yeilds smaller effect sizes than using a hierarchical approach. It is not necessary to find genes uniquely marking Tybe B samples because SLE samples can first be detected by high expression of OAS2, LY6E and ISG15 for example. Type A patients are then most easily identified among SLE samples by high expression of TYK2, DDX17 and ZAP70. The product of this script highlights genes which may then be used to mark samples as Type 0, B (high MZB1, TXNDC5, IGLC3) or C

# This was used to look for multiple pairwise rank-sum tests

# It applies Kruskal-Wallis followed by Dunn's post-test

# for simplicity samples in a group should be adjacent in the mastersheet

library(dunn.test) #must first install.packages("dunn.test")

par(mfrow=c(1,1))

rm(list = ls(all = TRUE)) ## Clear Workspace

file <- read.csv('./TCellTF/GED_AB.csv', colClasses = "character")

file <- file[1:6094,]

### Function which generates plot for one gene, e.g geneplot("IFIT3") ###

expPlot <- function (exp) {

x <- which(file$Symbol == exp)

expALL <- as.numeric(file[x,30:47])

expCON <- as.numeric(file[x,30:33])

expO <- as.numeric(file[x,34:36]) #groupO samples location

expA <- as.numeric(file[x,37:39])

expB <- as.numeric(file[x,40:44])

expC <- as.numeric(file[x,45:47])

medians <- sapply(list(expALL, expCON, expO, expA, expB, expC), median)

stdevs <- sapply(list(expALL, expCON, expO, expA, expB, expC), sd)

names(medians) <- c("Combined", "CON", "O", "A", "B", "C")

expdf <- data.frame(

Expression = c(expCON, expO, expA, expB, expC),

Group = factor(rep(c("CON", "O", "A", "B", "C"),

times=c(length(expCON), length(expO), length(expA), length(expB), length(expC))))

)

kw <- kruskal.test(Expression~Group, data=expdf)

result <- kw$p.value < 0.05

par(cex.lab=1.5)

plotMax <- 1.3*max(expCON, expO, expA, expB, expC)

bp <- barplot(medians, ylim=c(0,plotMax), ylab = "Expression (FPKM)",

cex.axis = 2, col=c("white","blue","green","yellow","orange","red"),

xlab= "Sample Type",

main = exp,cex.main=2,cex.names=2,

sub = paste("Kruskal-Wallis p< 0.05 : ", result))

arrows(bp, medians-stdevs, bp, medians + stdevs, bp, angle = 90, code = 3, lwd=3)

points(jitter(rep(bp[1,1],length(expALL)),amount=0.2),expALL, pch = 21, bg = "black")

points(jitter(rep(bp[2,1],length(expCON)),amount=0.2),expCON, pch = 21, bg = "black")

points(jitter(rep(bp[3,1],length(expO)),amount=0.2),expO, pch = 21, bg = "black")

points(jitter(rep(bp[4,1],length(expA)),amount=0.2),expA, pch = 21, bg = "black")

points(jitter(rep(bp[5,1],length(expB)),amount=0.2),expB, pch = 21, bg = "black")

points(jitter(rep(bp[6,1],length(expC)),amount=0.2),expC, pch = 21, bg = "black")

}

### Statistical Tests ###

kw_logical <- c() ## TRUE/FALSE vector for each exp, Kruskal-Wallis p<0.05 (takes time)

for (i in file$Symbol) {

x <- which(file$Symbol == i)

expALL <- as.numeric(file[x,30:47])

expCON <- as.numeric(file[x,30:33])

expO <- as.numeric(file[x,34:36])

expA <- as.numeric(file[x,37:39])

expB <- as.numeric(file[x,40:44])

expC <- as.numeric(file[x,45:47])

expdf <- data.frame(

Expression =c(expCON, expO,expA,expB,expC),

Group =factor(rep(c("CON", "O", "A", "B", "C"),

times=c(length(expCON), length(expO), length(expA), length(expB), length(expC))))

)

result <- kruskal.test(Expression~Group, data=expdf)$p.value < 0.05

kw_logical <- c(kw_logical, result)

}

kwTRUE <- file$Symbol[which(kw_logical == TRUE)] ## Genes where Kruskal-Wallis p<0.05

dunns <- data.frame() #for each exp with p < 0.05, create numeric vector for each pairwise comparison using Dunn's test

for (i in kwTRUE) {

x <- which(file$Symbol == i)

expALL <- as.numeric(file[x,30:47])

expCON <- as.numeric(file[x,30:33])

expO <- as.numeric(file[x,34:36])

expA <- as.numeric(file[x,37:39])

expB <- as.numeric(file[x,40:44])

expC <- as.numeric(file[x,45:47])

expdf <- data.frame(

Expression =c(expCON,expO,expA,expB,expC),

Group =factor(rep(c("CON", "O", "A", "B", "C"),

times=c(length(expCON), length(expO), length(expA), length(expB), length(expC))))

)

dunn <- dunn.test(expdf$Expression, expdf$Group, method = "bonferroni")

dunns <- rbind(dunns, round(dunn$P.adjusted, 3))

}

dunndf <- data.frame(kwTRUE, dunns)

names(dunndf) <- c("Gene", "BvA", "CvA", "CvB", "CONvA", "CONvB", "CONvC", "OvA", "OvB", "OvC", "OvCON")

### Analysis ###

sumdunn <- c(

sum(dunndf$BvA < 0.05),

sum(dunndf$CvA < 0.05),

sum(dunndf$CvB < 0.05),

sum(dunndf$CONvA < 0.05),

sum(dunndf$CONvB < 0.05),

sum(dunndf$CONvC < 0.05),

sum(dunndf$OvA < 0.05),

sum(dunndf$OvB < 0.05),

sum(dunndf$OvC < 0.05),

sum(dunndf$OvCON < 0.05))

names(sumdunn) <- c("BvA", "CvA", "CvB", "CONvA", "CONvB", "CONvC", "OvA", "OvB", "OvC", "OvCON")

## produces gene lists for each comparison for which Dunn test is significant

BvA <- dunndf$Gene[which(dunndf$BvA < 0.05)]

CvA <- dunndf$Gene[which(dunndf$CvA < 0.05)]

CvB <- dunndf$Gene[which(dunndf$CvB < 0.05)]

CONvA <- dunndf$Gene[which(dunndf$CONvA < 0.05)]

CONvB <- dunndf$Gene[which(dunndf$CONvB < 0.05)]

CONvC <- dunndf$Gene[which(dunndf$CONvC < 0.05)]

OvA <- dunndf$Gene[which(dunndf$OvA < 0.05)]

OvB <- dunndf$Gene[which(dunndf$OvB < 0.05)]

OvC <- dunndf$Gene[which(dunndf$OvC < 0.05)]

OvCON <- dunndf$Gene[which(dunndf$OvCON < 0.05)]

multiples <- data.frame() ## for given comparison, calculates and orders by ratio of medians

for (i in BvA) {

x <- which(file$Symbol == i)

expALL <- as.numeric(file[x,30:47])

expCON <- as.numeric(file[x,30:33])

expO <- as.numeric(file[x,34:36])

expA <- as.numeric(file[x,37:39])

expB <- as.numeric(file[x,40:44])

expC <- as.numeric(file[x,45:47])

medians <- sapply(list(expALL, expCON, expO, expA, expB, expC), median)

ratioBvA = round(max(medians[c(4,5)]) / min(medians[c(4,5)]), 2)

multiples <- rbind(multiples, ratioBvA)

}

BvAdf <- data.frame(BvA, multiples)

BvAdfordered <- BvAdf[order(-multiples),]

head(BvAdfordered, 20)

###

multiples <- data.frame()

for (i in CvA) {

x <- which(file$Symbol == i)

expALL <- as.numeric(file[x,30:47])

expCON <- as.numeric(file[x,30:33])

expO <- as.numeric(file[x,34:36])

expA <- as.numeric(file[x,37:39])

expB <- as.numeric(file[x,40:44])

expC <- as.numeric(file[x,45:47])

medians <- sapply(list(expALL, expCON, expO, expA, expB, expC), median)

ratioCvA = round(max(medians[c(4,6)]) / min(medians[c(4,6)]), 2)

multiples <- rbind(multiples, ratioCvA)

}

CvAdf <- data.frame(CvA, multiples)

CvAdfordered <- CvAdf[order(-multiples),]

head(CvAdfordered, 20)

###

multiples <- data.frame()

for (i in CvB) {

x <- which(file$Symbol == i)

expALL <- as.numeric(file[x,30:47])

expCON <- as.numeric(file[x,30:33])

expO <- as.numeric(file[x,34:36])

expA <- as.numeric(file[x,37:39])

expB <- as.numeric(file[x,40:44])

expC <- as.numeric(file[x,45:47])

medians <- sapply(list(expALL, expCON, expO, expA, expB, expC), median)

ratioCvB = round(max(medians[c(5,6)]) / min(medians[c(5,6)]), 2)

multiples <- rbind(multiples, ratioCvB)

}

CvBdf <- data.frame(CvB, multiples)

CvBdfordered <- CvBdf[order(-multiples),]

head(CvBdfordered, 20)

###

multiples <- data.frame()

for (i in CONvA) {

x <- which(file$Symbol == i)

expALL <- as.numeric(file[x,30:47])

expCON <- as.numeric(file[x,30:33])

expO <- as.numeric(file[x,34:36])

expA <- as.numeric(file[x,37:39])

expB <- as.numeric(file[x,40:44])

expC <- as.numeric(file[x,45:47])

medians <- sapply(list(expALL, expCON, expO, expA, expB, expC), median)

ratioCONvA = round(max(medians[c(2,4)]) / min(medians[c(2,4)]), 2)

multiples <- rbind(multiples, ratioCONvA)

}

CONvAdf <- data.frame(CONvA, multiples)

CONvAdfordered <- CONvAdf[order(-multiples),]

head(CONvAdfordered, 20)

###

multiples <- data.frame()

for (i in CONvB) {

x <- which(file$Symbol == i)

expALL <- as.numeric(file[x,30:47])

expCON <- as.numeric(file[x,30:33])

expO <- as.numeric(file[x,34:36])

expA <- as.numeric(file[x,37:39])

expB <- as.numeric(file[x,40:44])

expC <- as.numeric(file[x,45:47])

medians <- sapply(list(expALL, expCON, expO, expA, expB, expC), median)

ratioCONvB = round(max(medians[c(2,5)]) / min(medians[c(2,5)]), 2)

multiples <- rbind(multiples, ratioCONvB)

}

CONvBdf <- data.frame(CONvB, multiples)

CONvBdfordered <- CONvBdf[order(-multiples),]

head(CONvBdfordered, 20)

###

multiples <- data.frame()

for (i in CONvC) {

x <- which(file$Symbol == i)

expALL <- as.numeric(file[x,30:47])

expCON <- as.numeric(file[x,30:33])

expO <- as.numeric(file[x,34:36])

expA <- as.numeric(file[x,37:39])

expB <- as.numeric(file[x,40:44])

expC <- as.numeric(file[x,45:47])

medians <- sapply(list(expALL, expCON, expO, expA, expB, expC), median)

ratioCONvC = round(max(medians[c(2,6)]) / min(medians[c(2,6)]), 2)

multiples <- rbind(multiples, ratioCONvC)

}

CONvCdf <- data.frame(CONvC, multiples)

CONvCdfordered <- CONvCdf[order(-multiples),]

head(CONvCdfordered, 20)

###

multiples <- data.frame()

for (i in OvA) {

x <- which(file$Symbol == i)

expALL <- as.numeric(file[x,30:47])

expCON <- as.numeric(file[x,30:33])

expO <- as.numeric(file[x,34:36])

expA <- as.numeric(file[x,37:39])

expB <- as.numeric(file[x,40:44])

expC <- as.numeric(file[x,45:47])

medians <- sapply(list(expALL, expCON, expO, expA, expB, expC), median)

ratioOvA = round(max(medians[c(3,4)]) / min(medians[c(3,4)]), 2)

multiples <- rbind(multiples, ratioOvA)

}

OvAdf <- data.frame(OvA, multiples)

OvAdfordered <- OvAdf[order(-multiples),]

head(OvAdfordered, 20)

###

multiples <- data.frame()

for (i in OvB) {

x <- which(file$Symbol == i)

expALL <- as.numeric(file[x,30:47])

expCON <- as.numeric(file[x,30:33])

expO <- as.numeric(file[x,34:36])

expA <- as.numeric(file[x,37:39])

expB <- as.numeric(file[x,40:44])

expC <- as.numeric(file[x,45:47])

medians <- sapply(list(expALL, expCON, expO, expA, expB, expC), median)

ratioOvB = round(max(medians[c(3,5)]) / min(medians[c(3,5)]), 2)

multiples <- rbind(multiples, ratioOvB)

}

OvBdf <- data.frame(OvB, multiples)

OvBdfordered <- OvBdf[order(-multiples),]

head(OvBdfordered, 20)

###

multiples <- data.frame()

for (i in OvC) {

x <- which(file$Symbol == i)

expALL <- as.numeric(file[x,30:47])

expCON <- as.numeric(file[x,30:33])

expO <- as.numeric(file[x,34:36])

expA <- as.numeric(file[x,37:39])

expB <- as.numeric(file[x,40:44])

expC <- as.numeric(file[x,45:47])

medians <- sapply(list(expALL, expCON, expO, expA, expB, expC), median)

ratioOvC = round(max(medians[c(3,6)]) / min(medians[c(3,6)]), 2)

multiples <- rbind(multiples, ratioOvC)

}

OvCdf <- data.frame(OvC, multiples)

OvCdfordered <- OvCdf[order(-multiples),]

head(OvCdfordered, 20)

###

multiples <- data.frame()

for (i in OvCON) {

x <- which(file$Symbol == i)

expALL <- as.numeric(file[x,30:47])

expCON <- as.numeric(file[x,30:33])

expO <- as.numeric(file[x,34:36])

expA <- as.numeric(file[x,37:39])

expB <- as.numeric(file[x,40:44])

expC <- as.numeric(file[x,45:47])

medians <- sapply(list(expALL, expCON, expO, expA, expB, expC), median)

ratioOvCON = round(max(medians[c(2,3)]) / min(medians[c(2,3)]), 2)

multiples <- rbind(multiples, ratioOvCON)

}

OvCONdf <- data.frame(OvCON, multiples)

OvCONdfordered <- OvCONdf[order(-multiples),]

head(OvCONdfordered, 20)

expPlot("CD38")

We then looked directly at the files generated but in the prompt the output of the genes with the greatest expression difference will be listed.

**BioProject ID: PRJNA293549**

Submission of raw data is time consuming and involved repetitive naming of files and runs. Assemble the gzipped raw read data in a folder and calculate the checksum, likely using LSF syntax.

bsub -o check.out -q short -W 3:00 "md5sum *.gz > checklist.chk"

Then procede with sample logging at SRA. The following commands were used to upload the data, note the server name may change.

ftp ftp-private.ncbi.nlm.nih.gov

ftp> prompt n

Interactive mode off.

ftp> mput *.gz
